# Tackling similarities and differences in global practice guidelines for gastric cancer: a review on the latest Taiwan guidelines with Asia-Pacific, European and US guidelines

**DOI:** 10.1007/s10120-026-01753-8

**Published:** 2026-05-18

**Authors:** Chih-Chieh Yen, I-Chen Wu, Cheng-Chan Yu, Ming-Huang Chen, Nai-Jung Chiang, Wen-Liang Fang, Kuo-Hsing Chen, Chieh-Chang Chen, Tzu-Chan Hong, Yen-Cheng Chen, Ming-Wun Wong, De-Chuan Chan, I-Shu Chen, Yen-Yang Chen, Yueh-Wei Liu, Jiann-Ming Wu, Wen-Chi Chou, Chan-Keng Yang, Jun-Te Hsu, Ta-Sen Yeh, Li-Yuan Bai, Yi-Fang Chang, Yao-Yu Hsieh, Cheng-Ming Peng, Jaw-Yuan Wang, Hui-Hua Hsiao, Li-Tzong Chen, Kun-Ming Rau, Chia-Jui Yen, Jen-Shi Chen, Yan-Shen Shan

**Affiliations:** 1 Department of Oncology, National Cheng Kung University Hospital, College of Medicine, National Cheng Kung University, Tainan, Taiwan; 2 Division of Gastroenterology, Department of Internal Medicine, Kaohsiung Medical University Hospital, Kaohsiung Medical University, Kaohsiung, Taiwan; 3 Division of General Surgery, Department of Surgery, Taichung Tzu Chi Hospital, Taichung, Taiwan; 4 Department of Oncology, Taipei Veterans General Hospital, Taipei, Taiwan; 5 Department of Surgery, Taipei Veterans General Hospital, Taipei, Taiwan; 6 Department of Medical Oncology, National Taiwan University Cancer Center, Taipei, Taiwan; 7 Division of Gastroenterology and Hepatology, Department of Internal Medicine, National Taiwan University Hospital, Taipei, Taiwan; 8 Department of Medicine, National Taiwan University Cancer Center, Taipei, Taiwan; 9 Division of General Surgery, Department of Surgery, Hualien Tzu Chi Hospital, Hualien, Taiwan; 10 Department of Medicine, Hualien Tzu Chi Hospital, Hualien, Taiwan; 11 Division of General Surgery, Department of Surgery, Tri-Service General Hospital, Taipei, Taiwan; 12 Division of General Surgery, Department of Surgery, Kaohsiung Veterans General Hospital, Kaohsiung, Taiwan; 13 Division of Hematology-Oncology, Department of Internal Medicine, Kaohsiung Chang Gung Memorial Hospital, Kaohsiung, Taiwan; 14 Division of General Surgery, Department of Surgery, Kaohsiung Chang Gung Memorial Hospital, Kaohsiung, Taiwan; 15 Division of General Surgery, Department of Surgery, Far Eastern Memorial Hospital, New Taipei City, Taiwan; 16 Department of Hematology and Oncology, Chang Gung Memorial Hospital at Linkou and College of Medicine, Chang Gung University, Taoyuan, Taiwan; 17 Department of General Surgery, Chang Gung Memorial Hospital at Linkou and College of Medicine, Chang Gung University, Taoyuan, Taiwan; 18 Department of Hematology and Oncology, China Medical University Hospital, Taichung, Taiwan; 19 Division of Hematology and Oncology, Department of Internal Medicine, Mackay Memorial Hospital, Taipei, Taiwan; 20 Division of Hematology and Oncology, Department of Medicine, Taipei Medical University Shuang-Ho Hospital, New Taipei City, Taiwan; 21 Department of Surgery, Chung Shan Medical University Hospital, Taichung, Taiwan; 22 Division of Colorectal Surgery, Department of Surgery, Kaohsiung Medical University Hospital, Kaohsiung Medical University, Kaohsiung, Taiwan; 23 Division of Hematology and Oncology, Department of Internal Medicine, Kaohsiung Medical University Hospital, Kaohsiung Medical University, Kaohsiung, Taiwan; 24 Department of Hematology Oncology, E-Da Cancer Hospital, Kaohsiung, Taiwan; 25 Department of Surgery, National Cheng Kung University Hospital, College of Medicine, National Cheng Kung University, Tainan, Taiwan; 26 Center for Cancer Research, Kaohsiung Medical University, Kaohsiung, Taiwan

**Keywords:** Gastric cancer, Consensus, Biomarker, Management, Guidelines

## Abstract

**Background:**

Gastric cancer (GC) remains a global health burden. While international guidelines share consensus, variations exist in disputed issues. The *2025 Taiwan Consensus and Management Guidelines for Gastric Cancer* had just been released. We compared the key recommendations with established international guidelines.

**Methods:**

A multidisciplinary taskforce addressed key questions and recommendations using modified Delphi method and evidence-based approaches. The comparative review aimed to elucidate similarities and differences among guidelines from Taiwan, Japan, South Korea, China, Europe, and the US.

**Results:**

Guidelines converge on absolute endoscopic resection criteria for early GC but differ in extended indications and perioperative approaches for locally advanced disease. Heterogeneity exists in biomarker assessment protocols, cutoff thresholds, and companion diagnostics. For metastatic disease, consensus exists on anti-HER2, anti-VEGF, immunotherapy, and biomarker-driven strategies, though oligo-metastatic definitions and intraperitoneal chemotherapy indications remain controversial.

**Conclusion:**

Optimal GC management requires integrating global evidence with regional contexts. The comparative review addresses heterogeneity among international guidelines, which helps harmonize management strategies as therapeutic standards evolve.

**Supplementary Information:**

The online version contains supplementary material available at 10.1007/s10120-026-01753-8.

## Introduction

### Regional differences of gastric cancer in the world

Among gastrointestinal malignancies, gastric cancer (GC) represents a critical global health demand, ranking as the third most common cause of cancer mortality worldwide [1]. Geographic distribution reveals distinctive patterns, with Eastern Asia, Latin America, Eastern Europe, and Africa experiencing disproportionately high disease burden, underscoring critical ethnic and regional variations that must inform prevention and treatment approaches [2–5]. This persistent clinical challenge has prompted comprehensive practice guidelines aimed at optimizing diagnostic approaches and therapeutic interventions.

### Establishing the Taiwan consensus and management guidelines for gastric cancer

Data from Taiwan National Cancer Registry reveals that GC occupies the 10th for overall cancer incidence and 8th for mortality in Taiwan [6]. While recent trends suggest a modest shift toward earlier detection, Taiwan’s early-stage diagnosis rate falls substantially lower than comparable East Asian countries like Japan and South Korea, where early-stage identification ranges from 60% to 70% [7, 8]. Furthermore, epidemiological patterns in Taiwan demonstrate declining GC incidence over the past decades. This favorable trend likely reflects enhanced sanitation infrastructure combined with widespread *Helicobacter pylori* eradication [9, 10]. The therapeutic landscape has also evolved considerably in recent decades, driven by innovations in therapeutics [11]. This shift highlights a need for updated clinical guidance tailored to current and local practice.

Several international and validated guidelines from Asia-Pacific, Europe and US provide evidence-based information. However, variations in patient characteristics, healthcare systems, and disease patterns limit the direct applicability of these guidelines to every country. Despite considerable improvements, the quality of care and survival outcomes in Taiwan have not yet reached a comparable level to neighboring Eastern Asian counterparts such as Japan, South Korea or Singapore [12]. In the current practice paradigm, clinicians in Taiwan referred to relevant Asia-Pacific or Western guidelines routinely for clinical decision-making and management owing to the absence of a validated local reference in the past. Noticeably, national healthcare insurance agency in Taiwan constantly demands accountable local practice references, which affect the reimbursement of both diagnostic tools and therapeutics.

As a result, the Taiwan Gastric Cancer Association and its taskforce established the first guidelines for GC (*Taiwan Consensus and Management Guidelines for Gastric Cancer*,* 2025; 1st edition*), which have just been released. The present guidelines represent a collaborative effort among multiple disciplines, designed to propose evidence-based recommendations spanning the entire continuum of GC. Recommendation formulation integrates Taiwan’s specific regulatory framework while adhering to rigorous evidence-based principles, drawing upon internationally recognized practice guidelines to ensure global alignment and validity. However, despite efforts in counterbalancing differences among other reference guidelines when establishing the Taiwanese guidelines, variations still exist in some key disputed issues in GC.

### Tackling similarities and differences among global practice guidelines

Western-derived guidelines differ conceptually from Asia-Pacific ones due to distinctive tumor biology, care disparities, resource availability, therapeutic access, and regulatory policies [13, 14]. Addressing heterogeneity in established guidelines highlights disputed issues and emerging practice-changing discoveries.

Key controversies include: extending absolute criteria for endoscopic treatment of early GC; an increasingly recognized role of perioperative therapy in locally advanced resectable disease; biomarker assessment and the optimized workflows; debates on diverse therapeutic combinations; definitions in oligo-metastatic disease; indications for peritoneum-directed therapy. These issues not only highlight variations among established guidelines but also pave the way for recent developments in GC management.

The present review aimed to compare the recently released *Taiwan Consensus and Management Guidelines for Gastric Cancer (2025)* with the updated guidelines, including those from Japan, Europe, US, South Korea and China. Key controversies are discussed in a comparative manner. Together the narrative review affirmed various GC guidelines to ensure a global alignment and validity.

## Materials and methods

### Generating the Taiwan consensus and management guidelines for gastric cancer

A multidisciplinary consortium was established for generating the Taiwan guidelines, as described in Supplemental 1. This diverse representation ensured comprehensive coverage of clinical and research perspectives. The panel identified issues organized into three principal domains: [1] diagnosis and endoscopic treatment for early GC [2], management of locally advanced resectable GC, and [3] management of advanced, metastatic, or recurrent GC. Three representative panelists conducted preliminary screening to generate key questions (KQs) based on clinical significance, alignment with regulatory standards, quality of available evidence, and potential of reaching consensus. At each consensus meeting, panelists contemplated the existing evidence and attempted to achieve a consensus by rating using a modified Delphi method [15, 16]. The final recommendation statements were secured and released on 15/Sep/2025, fitting to the contemporary global evidence at the time point. The KQs, recommendation statements and rationale of reaching these statements from the *Taiwan Consensus and Management Guidelines for Gastric Cancer (2025)* were provided in Supplemental 2.

### Comparing the global guidelines for gastric cancer

Next, we incorporated the following significant publications spanning Asia-Pacific, Europe and US, which were reviewed based on the updated version mainly in English language: (1) Japanese Gastric Cancer Treatment Guidelines 2025 (7th edition) by Japanese Gastric Cancer Association (JGCA) [17], (2) Korean Practice Guidelines for Gastric Cancer 2024 by the Korean Gastric Cancer Association (KGCA) [18], (3) Clinical Guidelines for the Diagnosis and Treatment of Gastric Cancer 2023 by the Chinese Society of Clinical Oncology (CSCO) [19], (4) National Comprehensive Cancer Network Guideline for Gastric Cancer 2026 version 1.0 (NCCN) [20], and (5) European Society for Medical Oncology (ESMO) Guideline for Gastric Cancer 2022 and its living guideline (version 1.4; Sep/2024) [21, 22] plus the Pan-Asian adapted version (03/Feb/2024) [23]. 

### Evidence-based approaches and grading for recommendations

The formation of expert consensus and agreement is described in Supplemental 3, which was based on the Grading of Recommendations Assessment, Development, and Evaluation (GRADE) methodology. As a reference for physicians or healthcare professionals, the KQs, level of evidence, agreement and grade of recommendations are provided in parallel, which are summarized in Table 1.

**Table 1:** Key questions, evidence and agreement from the Taiwan consensus and management guidelines for gastric cancer (2025)

Key questions (KQs)		
*Screening, diagnosis and endoscopic treatment for early gastric cancer*		
KQ1-1		What are the recommended methods for early GC screening?
KQ1-2		Are stool- or blood-based assays recommended for pre-selecting the high-risk population?
KQ1-3		What is/are the required pre-procedure exam(s) for patients planning to receive EMR or ESD for early GC?
KQ1-4		If the pre-procedural exam for EMR/ESD suggests controversial perigastric lymph node metastasis, what is the recommended management?
KQ1-5		Could EMR/ESD be applied to patients with poorly differentiated or signet-ring cell histology?
KQ1-6		Could EMR or ESD be applied to patients with clinical T1b lesion?
KQ1-7		When the gastric lesion is resected in a piecemeal manner or the margins inadequate for a clear evaluation, could ESD be repeated?
KQ1-8		Could EMR/ESD be recommended for early GC treatment over surgery for unfit patients?
*Management of locally advanced resectable gastric cancer*		
KQ2-1		What is/are the recommended exam(s) for selecting patients with locally advanced GC planning to receive perioperative therapies?
KQ2-2		What is the recommended field of imaging for patients with locally advanced GC planning to receive perioperative therapies?
KQ2-3		How do we select patients with locally advanced GC for perioperative therapies?
KQ2-4		What are the ideal therapeutic regimens for perioperative therapies in locally advanced GC?
KQ2-5		Is prophylactic HIPEC recommended for patients with locally advanced M0 GC?
KQ2-6		What are the recommended postoperative adjuvant therapies for patients with locally advanced disease?
*Management of advanced, metastatic or recurrent GC*		
KQ3-1		For patients with advanced, metastatic or recurrent disease, what are the recommended biomarker assays prior to systemic treatment?
KQ3-2		For patients with advanced, metastatic or recurrent disease, is tumor-based MSI testing routinely required for these patients?
KQ3-3		How do we organize the recommended biomarker assays in patients with advanced, metastatic or recurrent disease?
KQ3-4		Is tumor- or plasma-based panelized sequencing assay recommended for patients with advanced, metastatic or recurrent disease?
KQ3-5		What are the recommended frontline therapeutic combinations for patients with advanced, metastatic or recurrent disease?
KQ3-6		What are the recommended subsequent therapeutic combinations for patients with advanced, metastatic or recurrent disease who have failed the frontline therapies?
KQ3-7		Is HIPEC recommended for patients with advanced, metastatic or recurrent disease?
KQ3-8		Is palliative gastrectomy recommended for patients with advanced, metastatic or recurrent disease?
KQ3-9		How do we define limited or oligo-metastatic disease which could potentially be converted to curative surgery?
*Level of evidence (LoE)*		
I	Publication(s) from at least one high-quality RCT or one high-quality meta-analysis	
II	Publication(s) from at least one medium to low-quality RCT or one medium to low-quality meta-analysis	
III	Publication(s) from at least one prospective cohort study or other observational studies	
IV	Publication(s) from at least one retrospective cohort study or other case-control studies	
V	Publication(s) from reviews, expert opinions or commentaries	
*Level of agreement (LoA)*		
*****	100% of voting agreement in the expert panel	
****	80–99% of voting agreement in the expert panel	
***	60–79% of voting agreement in the expert panel	
**	30–59% of voting agreement in the expert panel	
*	Absence of agreement	
*Grade of recommendation (GoR)*		
A	Strong evidence; strongly recommended	
B	Moderate evidence; moderately recommended	
C	Mild evidence; weakly recommended	
D	Insufficient evidence; insufficient to conclude	

GC, gastric cancer; EMR, endoscopic mucosal resection; ESD, endoscopic submucosal dissection; HIPEC, hyperthermic intraperitoneal chemotherapy; MSI, microsatellite stability; RCT, randomized controlled trial; LoE, level of evidence; LoA, level of agreement; GoR, grades of recommendation

*****full agreement; ****strong agreement; *** moderate agreement; ** weak agreement; * absence of agreement

We acknowledged that methodologies and terminologies were largely inconsistent among different guidelines (Supplemental 4). For example, the JGCA guidelines were mostly utilized GRADE-adopted method with several issues resolved by expert consensus. The KGCA guidelines followed the GRADE-adopted terminology and presented with a meta-analysis supporting each of the de novo issues. The NCCN guidelines incorporated unique *NCCN Categories of Evidence and Consensus* and entailed a “preferred” strategy instead of recommendation grades. Alternatively, the ESMO guidelines and derivatives had implemented the Infectious Diseases Society of America-United States Public Health Grading system, ESMO-Magnitude of Clinical Benefit Scale (ESMO-MCBS) and ESMO Scale for Clinical Actionability of molecular Targets (ESCAT) [24, 25]. The CSCO guidelines proposed an independent level of evidence and recommendation based on expert consensus.

## Diagnosis and endoscopic treatment for early gastric cancer

### Absolute criteria and pre-procedural exams for endoscopic resection of early gastric cancer

Endoscopic resection (ER) has been extensively implemented as a definitive treatment for early GC with recurrence risk and survival outcomes not inferior to conventional curative surgeries. Both endoscopic mucosal resection (EMR) and endoscopic submucosal dissection (ESD) have been proficiently considered as a standard treatment for early GC fitting prespecified criteria. The absolute criteria include well-differentiated mucosal lesions measuring 2 cm or less in diameter that lack ulcerative features. In general, the global guidelines share a consensus on absolute criteria for ER, rendering key differences in the extended or expanded indications for discussion.

Precise pre-procedural exams play an unreplaceable role in establishing appropriateness for ER under a curative intent. Computed tomography (CT) scan reported an acceptable diagnostic performance for differentiating mucosal/submucosal tumor from deeper invasion or adjacent lymph node involvement [26–28]. Endoscopic ultrasound (EUS) provides enhanced visualization of invasion depth, although results depend significantly on operator expertise [29, 30]. The KGCA, NCCN and ESMO guidelines had pinpointed that CT scan should be a required assessment to secure curability of ER, while EUS as a recommended tool in conjunction. However, Japanese and Chinese guidelines prioritized endoscopic assessment for early GC staging. Conventional white-light endoscopy aids depth estimation, while magnifying endoscopy combined with narrow-band imaging (ME-NBI) or chromoendoscopy allow experienced endoscopists to assess invasion depth and spreading pattern, leaving both CT scan and EUS as recommended but not required exams [31, 32]. The pre-procedural exams and absolute criteria for ER are summarized in Table 2.

**Table 2:** Absolute/extended criteria, pre-procedural exams and special considerations for endoscopic resection of early gastric cancer

Guidelines	Pre-procedural exam(s)	Absolute criteria	Extended criteria	Proceed to surgery	Special indications
TWGCA 2025 (KQ 1–3 to 1–8)	Required: CT scan Recommended: ME-NBI, chromo, EUS (LoE: IV; LoA: ***; GoR: C)	T1a, ≤ 20 mm, UL0, Diff	1) T1a, ≤ 20 mm, UL0, Undiff 2) T1b (SM1), ≤ 30 mm, VM0, Ly0, V0, Diff (LoE: IV; LoA: ***; GoR: C)	1) LN (+) 2) Piecemeal resection 3) Unclear margins (LoE: II; LoA: **; GoR: D)	Conditional for surgically unfit or elderly patients (LoE: IV; LoA: ****; GoR: C)
JGCA 2025	Recommended: ME-NBI, chromo, EUS, CT scan	For EMR or ESD: T1a, ≤ 20 mm, UL0, Diff For ESD: 1) T1a, > 20 mm, UL0, Diff 2) T1a, ≤ 30 mm, UL1, Diff 3) T1a, ≤ 20 mm, UL0, Undiff	1) Locally recurred lesion, T1a, Diff (eCuraC-1) 2) Resected lesion deemed as eCuraC-1	1) eCuraC-2 2) LN (+) 3) ≥T2 lesion	1) Conditional for surgically unfit or elderly patients 2) Informed consent after explaining risk of residual disease/LN metastasis
KGCA 2024	Required: CT scan Recommended: ME-NBI, chromo, EUS	For ER: T1a, ≤ 20 mm, UL0, Diff For ESD or surgery: 1) T1a, > 20 mm, UL0, Diff 2) T1a, ≤ 30 mm, UL1, Diff	T1a, ≤ 20 mm, UL0, Undiff	1) Non-curative resection 2) Piecemeal resection 3) LN (+) 4) LVI (+) 5) VM (+)	1) Conditional for surgically unfit or elderly patients 2) Informed consent after explaining risk of residual disease/LN metastasis
CSCO 2023	Recommended: ME-NBI, chromo, EUS, CT scan	For EMR/ESD: T1a, ≤ 20 mm, UL0, Diff For ESD: 1) T1a, > 20 mm, UL0, Diff 2) T1a, ≤ 30 mm, UL1, Diff 3) T1a, ≤ 20 mm, UL0, Undiff	1) Locally recurred lesion, T1a, Diff (eCuraC-1) 2) T1a, ≤ 20 mm, UL0, Undiff	1) Non-curative resection 2) LN (+)	Conditional for surgically unfit or elderly patients
NCCN 2026 (version 1)	Required: CT scan Recommended: EUS	≤ 20 mm, T1a, Diff, LVI (-), HM (-), VM (-) (Category 2 A)	No suggestions	1) Undiff lesions 2) LVI (+) 3) ≥T1b lesions 4) VM or HM (+) 5) > 20 mm (Category 2 A)	Binary allocation: either meets absolute criteria for ER or requires surgery
ESMO 2022, living guidelines 2024 (version 1.4) and Pan-Asian adapted	Required: CT scan Recommended: ME-NBI, chromo, EUS (Pan-Asian adapted)	T1a, ≤ 20 mm, UL0, Diff	1) T1a, > 20 mm, UL0, Diff 2) T1a, ≤ 30 mm, UL0, Diff (ESGE adapted)	1) Outside of any ER indications 2) Non-curative resection 3) LN (+)	Only under multidisciplinary discussion

ME-NBI, magnifying endoscopy combined with narrow-band imaging; EUS, endoscopic ultrasound; UL, ulceration; Diff, differentiated; Undiff, undifferentiated; ER, endoscopic resection; EMR, endoscopic mucosal resection; ESD, endoscopic submucosal dissection; VM, vertical margin; Ly, lymphatic invasion; V, vascular invasion; SM1, submucosal invasion 1; LVI, lymphovascular invasion; HM, horizontal margin; TWGCA, Taiwan Gastric Cancer Association; JGCA, Japan Gastric Cancer Association; KGCA, Korean Gastric Cancer Association; CSCO, Chinese Society of Clinical Oncology; NCCN, National Comprehensive Cancer Network; ESMO, European Society for Medical Oncology. LoE (II) publication(s) from at least one medium to low-quality RCT or one medium to low-quality meta-analysis; (IV) publication(s) from at least one retrospective cohort study or other case-control studies. LoA (**) 30–59%, (***) 60–79%, (****) 80–99% of voting agreement in the expert panel. GoR (C) weakly recommended; (D) insufficient to conclude

The present TWGCA guidelines endorsed the global guidelines and recognized lack of a universally superior modality. The panel concluded that the absolute criteria for ER is defined by well-differentiated mucosal lesions measuring 2 cm or less in diameter that lack ulcerative features (T1a, ≤ 20 mm, UL0, Diff), and that contrast-enhanced CT scan as the mandatory assessment prior to ER, while EUS or ME-NBI are adjunctive tools to secure the pre-procedural curability. (KQ 1-3)

### Extended criteria and special considerations for endoscopic resection of early gastric cancer

Emerging evidence has supported the extended or expanded criteria for ER. For instance, it has been broadened to include poorly differentiated and signet-ring cell histologies—specifically when tumors remain 2 cm or smaller, are limited to mucosa and are absent of ulceration or lymphovascular invasion [33–35]. Differentiated tumors over 2 cm of size or ulcerated tumors have also been discussed provided that they meet the appropriate histologic requirements for a curative resection [36]. En bloc and complete resection rates remain high even under these extended indications [37–40]. The fundamental extension reflects an increased acknowledgment of ER’s therapeutic efficacy.

For cT1b lesions, gastrectomy with lymph node dissection has been the standard of care due to risk of nodal involvement. However, in patients meeting the extended submucosal invasion criteria, defined as submucosal invasion < 500 μm, differentiated histology, no lymphovascular invasion, and tumor size ≤ 30 mm, the metastasis incidence was conditionally acceptable by 2–3% [34]. These findings indicate that while ESD is technically feasible for cT1b cases, its limited curative potential and higher nodal involvement warrant caution.

Considering piecemeal or R1 resection, while it is generally considered non-curative and should proceed to surgery, the 2022 European Society of Gastrointestinal Endoscopy guidelines supported that when lesions are limited to horizontal margin in the absence of high-risk features, risk of nodal involvement is within acceptable range. In these cases, repeated ESD could be an alternative to surgery [41]. The JGCA guidelines also proposed that eCuraC-1 (positive horizontal margin or piecemeal resection with otherwise curative features) could potentially be managed with repeated ESD when the risk and benefit between ER and surgery is carefully weighed [42]. In addition, for elderly or frail patients who are not candidates for surgery, ESD achieves over 90% disease-specific survival with low morbidity. Both Western and Asian large-scale cohort studies have confirmed a comparable efficacy of ESD, though not yet standard in all guidelines [43–46]. 

By consensus, the global guidelines have demonstrated a certain degree of extension to the absolute criteria for ER. The Asian-Pacific guidelines additionally adopted ER as an alternative for surgically unfit patients. The extended criteria and special considerations in summarized in Table 2. The present TWGCA guidelines shared an agreement with the global ones, while considering extending the criteria for cT1b disease only when tumors are limited to superficial submucosa (< 500 μm), size less than 30 mm, negative nodal or distal metastases and negative for lymphovascular invasion. In addition, the expert panel concluded that piecemeal or R1 resections should still be managed by surgery and reserve ESD only when surgical approach is not feasible. (KQ 1-4 to 1-8)

## Management of locally advanced resectable gastric cancer

### Selection criteria and suggested regimens as perioperative therapies for locally advanced resectable gastric cancer

Perioperative therapies for locally advanced resectable GC vary by region in the world. In Western countries, the fluorouracil, leucovorin, oxaliplatin, and docetaxel (FLOT) combination has emerged as the preferred perioperative regimen owing to its superiority confirmed by AIO-FLOT4 study [47]. Both NCCN and ESMO guidelines designated FLOT as perioperative treatment for suitable candidates.

Asian-Pacific studies have been exploring distinct chemotherapy protocols for their patient populations. In the Korean PRODIGY study, docetaxel, oxaliplatin, and S-1 (DOS) had been evaluated preoperatively with surgery followed by adjuvant S-1 monotherapy in cT2-3 N(+) or cT4 cases. This approach met its primary endpoint and yielded enhanced PFS and better resectability [48]. The Chinese RESOLVE trial compared perioperative S-1 plus oxaliplatin (SOX) against postoperative capecitabine plus oxaliplatin (CAPOX) in cT4aN(+) or cT4b tumors. The perioperative strategy produced superior disease-free and OS outcomes when perioperative SOX was compared with adjuvant CAPOX, while adjuvant SOX was non-inferior to adjuvant CAPOX [49, 50]. Conversely, the JCOG0501 study aimed to explore whether adding preoperative S-1/cisplatin improved outcomes versus upfront surgery with adjuvant S-1 in Borrmann type 4 or large type 3 lesions (≥ 8 cm). This neoadjuvant approach did not confer survival advantages in this challenging population [51]. Another Japanese trial evaluated S-1/cisplatin as preoperative treatment specifically for those with para-aortic lymph node involvement or bulky lymphadenopathy [52]. Although results suggested benefit in these high-risk cases, the JGCA guidelines did not endorse particular neoadjuvant or perioperative protocols in this setting.

The KEYNOTE-585 study confirmed the efficacy of pembrolizumab plus chemotherapy compared with chemotherapy alone. Although pathological complete response rates were significantly higher in the pembrolizumab arm, the trial did not achieve its primary survival endpoint [53]. In contrast, the MATTERHORN trial demonstrated that combining durvalumab with FLOT (D-FLOT) yielded substantial improvements in survival outcomes and favorable tumor regression [54]. Furthermore, the survival benefits were held constant across different ethnic groups with an acceptable tolerability to D-FLOT [55]. These findings are anticipated to change current treatment approaches.

In summary, given the existing evidence and variations between Western and Asian guidelines, the TWGCA expert panel concluded that patients who are fit for systemic treatments with either cT2-3 plus N(+) M0 or cT4 plus any N M0 disease are appropriate candidates to receive perioperative therapies. The selection criteria and suggested regimens as perioperative therapies for locally advanced resectable GC are summarized in Table 3. In the present TWGCA guidelines, FLOT or DOS is the backbone of perioperative therapies. Doublet chemotherapy is an alternative for patients who are ineligible for triplet regimens. These recommendations contemplated the incorporation of other therapeutics, such as PD-1/PD-L1 inhibitors given the emerging evidence. (KQ 2-3, 2-4)

**Table 3:** Selection criteria and suggested regimens as perioperative therapies for locally advanced resectable gastric cancer

Guidelines	Selection criteria	Recommended regimens	Notes
TWGCA 2025 (KQ 2–3, 2–4)	Resectable disease: 1) cT2-3 N(+)M0 2) cT4AnyNM0 (LoE: I; LoA: ****; GoR: B)	Recommended: Triplet: FLOT or DOS^a^ Alternatives: 1) Doublet: Plt-F 2) Addition of PD-1/PD-L1 inhibitor(s)^b^ to triplet chemotherapy (LoE: I; LoA: ****; GoR: B)	
JGCA 2025	No clear recommendations Selected: only for bulky N disease^c^	No clear recommendations For bulky N disease: Cisplatin/S-1	
KGCA 2024	Resectable stage II-III disease: No clear definitions	DOS, SOX	
CSCO 2023	Resectable stage III disease: 1) cT3-T4aN(+)M0 2) cT4bAnyNM0	DOS, FLOT, SOX	
NCCN 2026 (version 1)	Resectable disease: ≥cT2AnyNM0	Preferred: 1) FLOT (Category 1) 2) FLOT + Durvalumab (PD-L1 CPS ≥ 1 or TAP ≥ 1%) (Category 1) Alternatives: Cisplatin-F	MSI-H or dMMR tumors: 1) Dostarlimab 2) Nivolumab/ipilimumab followed by nivolumab 3) Pembrolizumab 4) Tremelimumab/durvalumab
ESMO 2022, living guidelines 2024 (version 1.4)	ESMO 2022 and living guideline 2024: Resectable stage IB -III disease: ≥cT2AnyNM0	ESMO 2022 and living guideline 2024: Triplet: FLOT (Grade A) Doublet: Plt-F (Grade A) Alternatives: ECF/ECX	
ESMO Pan-Asian adapted 2024	Selected for stage IB -III disease	Triplet: FLOT or DOS (Grade A) Doublet: Plt-F (Grade A)	

a. platinum-fluoropyrimidine-taxane triplet protocols other than FLOT or DOS should be weighed by local guidance and multidisciplinary discussions

b. Addition of a PD-1/PD-L1 inhibitor to triplet chemotherapy is based on emerging evidence but not fully established in routine practice, which should be weighed by local guidance and multidisciplinary discussions

c. as defined by (1) supra-pancreatic lymph nodes ≥ 30 mm, (2) two adjacent lymph nodes ≥ 15 mm or (3) para-aortic lymph nodes ≥ 10 mm in diameter (limited to No. 16a2/b1 region)

TWGCA, Taiwan Gastric Cancer Association; JGCA, Japan Gastric Cancer Association; KGCA, Korean Gastric Cancer Association; CSCO, Chinese Society of Clinical Oncology; NCCN, National Comprehensive Cancer Network; ESMO, European Society for Medical Oncology. FLOT, 5-fluorourail/leucovorin/oxaliplatin/docetaxel; Plt-F; platinum plus fluoropyrimidine; DOS, S-1/oxaliplatin/docetaxel; SOx, S-1/oxaliplatin; CPS, combined proportional score; TAP, tumor area positivity; MSI-H, microsatellite instability-high; dMMR, deficient mismatch repair; ECF, epirubicin/cisplatin/5-fluorourail; ECX, epirubicin/cisplatin/capecitabine. LoE (I) publication(s) from at least one high-quality RCT or one high-quality meta-analysis. LoA (****) 80–99% of voting agreement in the expert panel. GoR (B) moderately recommended

### Suggested postoperative adjuvant chemotherapy or concurrent chemoradiotherapy for patients who have received a curative gastrectomy

Radical gastrectomy plus D2 lymph node dissection constitutes the standard curative treatment for localized GC. However, without adjuvant systemic therapy, patients with advanced tumors face considerable relapse and progression. In the ACTS-GC trial, researchers randomized 1059 stage II (excluding T1) or III GC patients (per *Japanese Classification of Gastric Adenocarcinoma*, 2nd English edition) who had undergone D2 gastrectomy [56]. Participants were assigned to either 12 months of S-1 monotherapy or surveillance alone, with findings demonstrating marked survival advantages in the S-1 arm. Likewise, the CLASSIC study enrolled participants across South Korea, China, and Taiwan with stage II-IIIB disease (per AJCC 6th edition staging) who had completed D2 gastrectomy [57]. The trial compared 6 cycles of CAPOX against surveillance alone, yielding outcomes comparable to those observed in ACTS-GC. These landmark trials collectively established S-1 or CAPOX as valid adjuvant options for stage II-III GC patients after D2 surgery, which were well-demonstrated on the Asian-based guidelines.

Additionally, the JACCRO GC-07 study evaluated docetaxel plus S-1 in high-risk patients. Among stage III cases (per *Japanese Classification of Gastric Adenocarcinoma*, 3rd English edition), the doublet regimen yielded significantly better results than S-1 monotherapy [58]. Consistent findings also emerged from Korea’s ARTIST-2 trial, which enrolled stage II-III node-positive patients and confirmed that SOX outperformed S-1 monotherapy [59]. Likewise, in the China RESOLVE trial, adjuvant SOX was shown to be non-inferior to CAPOX, offering a substantial survival improvement in patients with cT4aN(+) or cT4b tumors [50]. On the other hand, based on phase III trials such as CRITICS, ARTIST and ARTIST2, the strength of adjuvant concurrent chemoradiotherapy (CCRT) has gradually dampened, leaving only the Western, KGCA and CSCO guidelines endorsed a limited suggestion if R1 resection or D1 dissection is achieved [59–61]. The role of adjuvant CCRT is considered less significant in Asia since most countries had adopted D2 dissection as surgical routine. In addition, the ATTRACTION-05 trial evaluated the role of adding nivolumab to adjuvant chemotherapy in stage III patients. The study did not reach its recurrence-free survival endpoint, though exploratory analyses indicated positive effects in those with stage IIIC tumors [62]. 

In summary, the TWGCA expert panel endorsed that adjuvant S-1 or CAPOX is recommended for pStage II disease, while S-1-based doublets, CAPOX, or FOLFOX are recommended for pStage III disease, leaving no clear suggestions for adjuvant CCRT (Table 4). (KQ 2-6)

**Table 4:** Suggested postoperative adjuvant chemotherapy or concurrent chemoradiotherapy for patients who have received a curative gastrectomy

Guidelines	Selection	Recommended chemotherapy	Recommended CCRT	Notes
TWGCA 2025 (KQ 2–6) (LoE: II; LoA: ***; GoR: B)	pStage II, pStage III	pStage II: S-1, CAPOX pStage III: CAPOX, FOLFOX, SOX, DS	No recommendations	
JGCA 2025	pStage II, pStage III	pStage II: S-1, CAPOX pStage III: DS, CAPOX, SOX	No recommendations	
KGCA 2024	pStage II, pStage III	pStage II: S-1, CAPOX pStage III: CAPOX	No recommendations	
CSCO 2023	pStage II, pStage III	pStage II: S-1, CAPOX pStage III: CAPOX, SOX, DS	Conditional: only for < D2 dissected or R1 resection 5-FU/capecitabine + cisplatin-based CRT	
NCCN 2026 (version 1)	pStage IB-III 1) ≥T2N0 2) not receive perioperative chemotherapy	Preferred: CAPOX, FOLFOX (Category 1) Alternatives: Fluoropyrimidine-based regimens	Conditional: only for < D2 dissected or R1 resection 5-FU/capecitabine-based CRT	
ESMO 2022, living guidelines 2024 (version 1.4)	pStage IB-III 1) ≥T2N0 2) not receive perioperative chemotherapy	ESMO 2022 and living guideline 2024: Doublet: (Grade A) 1) Fluoropyrimidine + oxaliplatin 2) Fluoropyrimidine + docetaxel DS, CAPOX	Conditional: only for < D2 dissected or R1 resection	
ESMO Pan-Asian adapted 2024	Same above	pStage II: S-1, CAPOX pStage III: DS, CAPOX	Same above	MSI-H: Chemotherapy should be carefully considered

TWGCA, Taiwan Gastric Cancer Association; JGCA, Japan Gastric Cancer Association; KGCA, Korean Gastric Cancer Association; CSCO, Chinese Society of Clinical Oncology; NCCN, National Comprehensive Cancer Network; ESMO, European Society for Medical Oncology; FOLFOX, 5-FU/leucovorin/oxaliplatin; CAPOX, capecitabine/oxaliplatin; DS, S-1/docetaxel; SOX, S-1/oxaliplatin; MSI-H, microsatellite instability-high. LoE (II) publication(s) from at least one medium to low-quality RCT or one medium to low-quality meta-analysis. LoA (***) 60–79%. GoR (B) moderately recommended

## Management of advanced, metastatic, or recurrent gastric cancer

### Proposed pre-treatment biomarker assessment and its protocol

Biomarker assessments in patients with advanced or metastatic GC play a crucial role in selecting optimal treatment strategies. Immunohistochemistry (IHC)-based testing offers practical advantages due to its rapid turnaround time and cost-effectiveness. Several biomarkers have demonstrated substantial clinical value, including human epidermal growth factor receptor-2 (HER2) overexpression, immune checkpoint proteins such as PD-1/PD-L1 and claudin isoform 18.2 (CLDN18.2) [18, 20, 23]. 

However, variations exist in testing methodologies, antibody selection for staining, interpretation criteria, and positivity thresholds. While some guidelines have proposed the use of combined positive scores (CPS) for PD-L1, no single cutoff value has been uniformly established. The suggested thresholds for PD-L1 expression spanned across all comers to patients with either CPS ≥ 1, ≥5 or ≥ 10, depending on the immune checkpoint inhibitors (ICIs) and clinical scenarios. The regulatory approval of ICIs had also been influenced by the selected PD-L1 antibody clones as companion diagnostic tools. For instance, PD-L1 Dako 22c3 is selected for introducing pembrolizumab, while Dako 28 − 8 is suggested for nivolumab. The Tumor Area Positivity (TAP) scoring with Ventana SP263 has additionally been adopted for introducing tislelizumab into systemic therapy. Although inter-assay heterogeneity exists across different PD-L1 testing clones, an acceptable concordance is reached in defining PD-L1 positive population (CPS ≥ 1 or TAP ≥ 1%) and may be considered interchangeable [63, 64]. For CLDN18.2, a general recommendation has linked specific anti-CLDN18.2 therapeutics to particular expression thresholds defined in clinical trials.

Microsatellite instability (MSI) assessment depicts the length variations in the typical repetitive sequences of marker genes and is regarded as a phenotypic feature of mismatch repair deficiency (dMMR). Consequently, a small proportion of tumors (< 5%) may exhibit discordance between MSI status and MMR expression [65]. If the cost- and time-effectiveness are not considered, an overall evaluation covering both MSI and MMR status could be suggested as a complementary approach.

Comprehensive genomic profiling (CGP) through selected gene panel uncovers actionable genetic alterations in cases where standard biomarker testing yields negative results. Tissue- or blood-based molecular analyses offer complementary information, capturing genomic variations across different time points and tumor locations [66]. Various molecular targets have been matched with corresponding targeted therapies, including high tumor mutational burden (TMB), B-Raf proto-oncogene (BRAF) mutations, fusions involving neurotrophic tyrosine receptor kinase (NTRK) or rearranged during transfection proto-oncogene (RET), fibroblast growth factor receptor 2 (FGFR2) alterations, and mesenchymal-epithelial transition factor (c-MET) amplifications [67]. Nevertheless, these aberrations occur in a relatively small proportion, with detection frequencies considerably lower than those of IHC-detectable biomarkers. As such, CGP is generally not positioned as a first-line testing approach. Additionally, while circulating tumor DNA (ctDNA) analysis from blood sampling has introduced novel insight for monitoring and clinical decision-making, existing international guidelines have not yet adopted routine liquid biopsy protocols beyond investigational purposes.

Global guidelines have reached agreement on the use of IHC-based testing, identifying HER2, PD-L1, MSI/MMR, and/or CLDN18.2 as key biomarkers. CGP serves as an additional testing to be pursued when conventional assessments fail to identify actionable targets. The utility of blood-based molecular testing remains limited to investigational contexts. Proposed pre-treatment biomarker assessment and its protocol are summarized in Table 5. The TWGCA guidelines had adopted this consensus framework, advocating for IHC-based biomarker testing at the outset of treatment planning, while positioning CGP or ctDNA testing as a secondary or investigational tool. (KQ 3-1 to 3-4)

**Table 5:** Proposed pre-treatment biomarker assessment for patients with metastatic, recurrent or advanced gastric cancer

Guidelines	Biomarkers								Requirements		
	HER2	PD-L1	MSI/MMR	CLDN18.2	EBV	TMB	NGS panel	Others	Timing	Tissue	Liquid biopsy
TWGCA 2025 (KQ 3 − 1 to 3–4)	Required IHC (LoE: I; LoA: *****; GoR: A)	Required IHC (CPS, cutoffs by assay) (LoE: I; LoA: *****; GoR: A)	Required MMR: IHC Optional MSI: PCR (LoE: II; LoA: ****; GoR: B)	Required IHC (LoE: I; LoA: *****; GoR: A)	NIL	Optional NGS (LoE: V; LoA: **; GoR: C)	Optional (LoE: V; LoA: **; GoR: C)	NIL	For IHC: Frontline; SIM (LoE: II; LoA: *****; GoR: A)	Standard; additional for NGS	Supplemental if insufficient tissue (LoE: V; LoA: **; GoR: C)
JGCA 2025	Required IHC	Required IHC (CPS)	Required MMR: IHC MSI: PCR	Required IHC	NIL	NIL	NIL	NIL	For IHC: Frontline; SIM	Standard	NIL
KGCA 2024	Required IHC	Required IHC (CPS, cutoffs by assay)	Required MMR: IHC MSI: PCR	Emerging IHC	Optional ISH for EBER	Optional NGS	Optional	NTRK, RET, BRAF	For IHC: Frontline; SIM	Standard; additional for NGS	NIL
CSCO 2023	Required IHC	Required IHC (CPS or TAP; cutoffs by assay)	Required MMR: IHC MSI: PCR	Emerging IHC	Optional ISH for EBER	Optional NGS	Optional	NTRK, RET, BRAF	For all: HER2, MSI/MMR For ICI planning: TMB, PD-L1	Standard; additional for NGS	genotyping for HER2 resistance
NCCN 2026 (version 1)	Required IHC	Required IHC (CPS, cutoffs by assay)	Required MMR: IHC MSI: PCR or NGS	Emerging IHC	Optional ISH for EBER	Optional NGS	Optional Optional	NTRK, RET, BRAF	For IHC: Frontline; SIM MSI for all	Standard; additional for NGS	Supplemental if insufficient tissue
ESMO 2022, living guidelines 2024 (version 1.4) and Pan-Asian adapted	Required IHC	Required IHC (CPS, cutoffs by assay)	Required MMR: IHC MSI: PCR	ESMO 2022, living guidelines 2024: Emerging Pan-Asian adapted: Strong recommended IHC	NIL	NIL	NIL	NIL	For all: HER2, MSI/MMR, PD-L1 CLDN18.2 if available	Standard	NIL

HER2, human epidermal growth factor receptor-2; PD-L1, programmed death ligand-1; MSI/MMR, microsatellite instability/mismatch repair; CLDN18.2, claudin18.2; EBV, Epstein-Bar virus; TMB, tumor mutation burden; NGS, next generation sequencing; TWGCA, Taiwan Gastric Cancer Association; JGCA, Japan Gastric Cancer Association; KGCA, Korean Gastric Cancer Association; CSCO, Chinese Society of Clinical Oncology; NCCN, National Comprehensive Cancer Network; ESMO, European Society for Medical Oncology; IHC, immunohistochemistry; CPS, combined proportional score; TAP, tumor area positivity; ICI, immune checkpoint inhibitor; PCR, polymerase chain reaction; NIL, not mentioned; SIM, simultaneous. LoE (I) publication(s) from at least one high-quality RCT or one high-quality meta-analysis; (II) publication(s) from at least one medium to low-quality RCT or one medium to low-quality meta-analysis; (V) publication(s) from reviews, expert opinions or commentaries. LoA (**) 30–59%, (****) 80–99%, (*****) 100% of voting agreement in the expert panel. GoR (A) strongly recommended; (B) moderately recommended; (C) weakly recommended

### Suggested regimens for frontline systemic therapies

The integration of ICIs and targeted therapeutics alongside chemotherapy has fundamentally transformed management approaches for patients with advanced, metastatic, or recurrent GC. In HER2-positive tumors, the ToGA study established that combining trastuzumab with chemotherapy yielded substantial improvements in OS, positioning HER2-targeted therapy as the benchmark [68]. Building upon this foundation, the KEYNOTE-811 trial assessed the addition of pembrolizumab to trastuzumab-chemotherapy and revealed substantial gains in ORR and PFS. These benefits were particularly prominent in tumors exhibiting PD-L1 positivity, as defined by PD-L1 CPS ≥ 1 with Dako 22c3 assay, thereby establishing triple-modality therapy as the preferred strategy for patients whose tumors demonstrate both HER2 and PD-L1 positivity [69, 70]. 

Similarly, for HER2-negative disease, the KEYNOTE-859 study established that incorporating pembrolizumab with chemotherapy produced significant OS improvements [71]. While these survival advantages were evident across the entire patient population, they demonstrated the greatest benefit in patients with PD-L1 CPS ≥ 10 (Dako 22c3). These findings ultimately resulted in regulatory approval for the pembrolizumab-chemotherapy combination.

Nivolumab represented another pioneering PD-1 inhibitor. Data from the CheckMate-649 trial revealed that incorporating nivolumab into chemotherapy yielded superior OS [72]. Although survival improvements were observed throughout the entire cohort irrespective of PD-L1 status, patients with PD-L1 CPS ≥ 5 (Dako 28 − 8) derived a more prominent clinical benefit. In contrast, the East Asian-limited ATTRACTION-4 trial evaluated a similar approach. While the results failed to meet its primary endpoint, it nonetheless demonstrated relevant improvements in PFS when nivolumab was added to chemotherapy [73]. 

Of note, the RATIONALE-305 trial assessed tislelizumab combined with chemotherapy against chemotherapy alone. This study successfully met its primary endpoints, demonstrating statistically significant OS gains in unselected patients [74]. Consistent with previous observations, patients whose tumors exhibited higher PD-L1 expression, as determined through the TAP system (Ventana SP263), experienced the most substantial therapeutic advantages. Additionally, results from the ORIENT-16 trial indicated that incorporating sintilimab into chemotherapy prolonged survivals [75]. Nevertheless, access to these PD-1/PD-L1 blocking agents varies by geographic region, and their inclusion in clinical practice guidelines remains inconsistent across different international guidelines. Taken together, global treatment guidelines still exhibit considerable heterogeneity regarding whether to incorporate PD-L1 expression as a uniform indicator and which cutoff values are best suited in selecting candidates for ICI therapy.

Furthermore, the phase III SPOTLIGHT and GLOW trials established the therapeutic value of zolbetuximab-chemotherapy combinations in patients with CLDN18.2-expressing HER2-negative advanced GC/GEJC. CLDN18.2 positivity was defined by the presence of moderate-to-strong membranous immunoreactivity in a minimum of 75% of tumor cells, as evaluated using the Ventana RxDx 43–14 A assay. Collective data had confirmed the survival benefit of incorporating zolbetuximab into chemotherapy for managing CLDN18.2-positive disease [76, 77]. 

Despite MSI-H or dMMR tumors constitute only a minor subset of all cases, revitalizing antitumor immunity through ICIs has demonstrated significant promise. Post-hoc subgroup analyses derived from CheckMate-649, KEYNOTE-062, and KEYNOTE-859 trials have consistently pinpointed marked therapeutic benefits when ICIs were combined with chemotherapy. Additionally, the phase II KEYNOTE-158 trial evaluated pembrolizumab in patients harboring MSI-H/dMMR tumors, yielding encouraging efficacy even in the heavily-pretreated patients [78]. However, considerable divergence exists regarding whether ICI monotherapy or ICI-chemotherapy should be prioritized as initial treatment. Western guidelines typically advocate for these strategies as tumor-agnostic frontline interventions, whereas Asia-Pacific ones tend to reserve them as second-line alternatives.

Regarding the incorporation of ICIs or targeted agents into chemotherapy frameworks, international guidelines demonstrate a general concordance. However, standardization of biomarker thresholds and assessment methodologies remain elusive. For example, while the JGCA guidelines acknowledged the therapeutic value of ICIs, they refrained from establishing specific cutoff criteria. Similarly, the KGCA, NCCN, and ESMO guidelines emphasized that PD-L1 expression as an indicator for identifying patients most likely to achieve benefits from chemoimmunotherapy. The CSCO guidelines took an additional step by including ICI agents with China-specific availability and delineating their corresponding companion diagnostics. Recognizing the heterogeneity among international guidelines and regional differences in drug accessibility, the TWGCA expert panel concluded biomarker-guided treatment algorithms for treatment-naive patients. These recommendations are summarized in Table 6. (KQ 3-5).

**Table 6:** Suggested regimen as frontline systemic therapies for patients with metastatic, recurrent or advanced gastric cancer

Guidelines	HER2-positive		HER2-negative		Others
TWGCA 2025 (KQ 3–5) (LoE: I; LoA: *****; GoR: A)	**PD-L1 CPS ≥1**^**a**^: Pembro + T + Plt-F^d^	**PD-L1 CPS < 1**: T + Plt-F^d^	**PD-L1 CPS ≥1**^**a**^: PD-1/PD-L1 inhibitor^e^ + Plt-F^d^ **PD-L1 CPS ≥5**^**a**^: PD-1/PD-L1 inhibitor^e^ + Plt-F^d^	**PD-L1 CPS < 1** or **CLDN18.2-negative** or **unselected**: Plt-F^d^	**dMMR/MSI-H**: Nivo + Ipi Pembro PD-1/PD-L1 inhibitor^e^ + Plt-F^d^
			**CLDN18.2-positive**^**b, c**^: Zolbe + Plt-F^d^		
JGCA 2025	XP/CAPOX/SP/SOX + T		**CLDN18.2-positive**^**b, c**^: CAPOX/FOLFOX + Zolbe CAPOX/FOLFOX + Nivo/Pembro^f^ FP + Pembro^f^ SOX + Nivo^f^	**CLDN18.2-negative**^**c**^: CAPOX/FOLFOX + Nivo/Pembro^f^ FP + Pembro^f^ SOX + Nivo^f^	dMMR/MSI-H for second-line
KGCA 2024	**PD-L1 CPS ≥1**^**a**^: FP/XP/CAPOX/FOLFOX + Pembro + T	**PD-L1 CPS < 1**: FP/XP/CAPOX/FOLFOX + T	**PD-L1 CPS ≥1**^**a**^: FP/CAPOX + Pembro **PD-L1 CPS ≥5**^**a**^: FOLFOX/CAPOX + Nivo	**PD-L1 CPS < 1** or **CLDN18.2-negative** or **unselected**: Plt-F^d^	dMMR/MSI-H for second-line
			**CLDN18.2-positive**^**b, c**^: Zolbe + FOLFOX/CAPOX		
CSCO 2023	FP/XP/CAPOX/FOLFOX + T (Grade I)		**PD-L1 CPS ≥5**^**a**^: FOLFOX/CAPOX + Nivo FOLFOX/CAPOX + Sinti **PD-L1 TAP ≥ 5%**^**a**^: CAPOX + Tis (Grade I)	**PD-L1 unselected**: Plt-F^d^ Taxane-F (Grade I)	**dMMR/MSI-H**: Pembro (Grade II)
NCCN 2026 (version 1)	**PD-L1 CPS ≥1**^**a**^: FP/XP/FOLFOX/CAPOX + Pembro + T (Category 1)	**PD-L1 CPS < 1**: FP/XP/CAPOX/FOLFOX + T	**PD-L1 CPS ≥1**^**a**^: PD-1/PD-L1 inhibitor^e^ + FP/XP/CAPOX/FOLFOX **PD-L1 CPS ≥5**^**a**^: PD-1/PD-L1 inhibitor^e^ + FP/XP/CAPOX/FOLFOX (Category 1)	**PD-L1 CPS < 1** or **CLDN18.2-negative** or **unselected**: FP/XP/CAPOX/FOLFOX	**dMMR/MSI-H**: Pembro Dostarlimab Nivo + Ipi FOLFOX/CAPOX + Nivo/Pembro
			**CLDN18.2-positive**^**b, c**^: Zolbe + Plt-F^d^		
ESMO 2022, living guidelines 2024 (version 1.4)	**PD-L1 CPS ≥1**^**a**^: Pembro + T + Plt-F	**PD-L1 CPS < 1**: T + Plt-F	**PD-L1 CPS ≥5**^**a**^: PD-1/PD-L1 inhibitor^f^ + Plt-F^d^ **PD-L1 CPS 1-4**^**a**^: PD-1/PD-L1 inhibitor^f^ + Plt-F^d^	**PD-L1 CPS < 1** or **CLDN18.2-negative** or **unselected**: Plt-F^d^ + docetaxel (selective)	**dMMR/MSI-H**: Plt-F + Nivo/Pembro
			**CLDN18.2-positive**^**b, c**^: FOLFOX/CAPOX + Zolbe		
ESMO Pan-Asian adapted	Same above		**PD-L1 CPS ≥1**^**a**^: Plt-F^d^ + Pembro **PD-L1 CPS ≥5**^**a**^: Plt-F^d^+ Nivo **PD-L1 CPS ≥ 10**^**a**^: Plt-F^d^ + Nivo/Pembro	**PD-L1 CPS < 1** or **CLDN18.2-negative** or **unselected**: Plt-F^d^	**MSI-H**: Plt-F + Nivo/Pembro
			**CLDN18.2-positive**^**b, c**^: Zolbe + Plt-F^d^		

a. PD-L1 assays are tailored to the companion PD-1/PD-L1 inhibitors: pembrolizumab (pharmDx Dako 22C3; Agilent Technologies, Carpinteria, CA, USA) or nivolumab (pharmDx Dako 28 − 8; Dako, Santa Clara, CA, USA). PD-L1 TAP score is an alternative and specifically utilized for tislelizumab with a cut-off of 5% (Ventana SP263 RxDx; Roche, Tucson, AZ, USA)

b. at least 75% of tumor cells with membrane staining (Ventana 43–14 A)

c. irrespective of PD-L1 status

d. including oxaliplatin or cisplatin plus one of the fluoropyrimidines, such as capecitabine, S-1 or fluorouracil

e. including pembrolizumab, nivolumab or tislelizumab

f. The expression level of PD-L1 and the presence of MSI-high/dMMR could be considered for applying nivolumab or pembrolizumab. However, no specific threshold was provided

HER2, human epidermal growth factor receptor-2; PD-L1, programmed death ligand-1; MSI/MMR, microsatellite instability/mismatch repair; CLDN18.2, claudin18.2; TWGCA, Taiwan Gastric Cancer Association; JGCA, Japan Gastric Cancer Association; KGCA, Korean Gastric Cancer Association; CSCO, Chinese Society of Clinical Oncology; NCCN, National Comprehensive Cancer Network; ESMO, European Society for Medical Oncology;.Plt-F, platinum-fluoropyrimidines; Nivo, nivolumab; Ipi, ipilimumab; Pembro, pembrolizumab; X, capecitabine; P, cisplatin; S, S-1; OX, oxaliplatin; T, trastuzumab; F, fluorouracil. LoE (I) publication(s) from at least one high-quality RCT or one high-quality meta-analysis. LoA (*****) 100% of voting agreement in the expert panel. GoR (A) strongly recommended

Bold labeling represents the subheadings or highlighted conditions in each box of treatment combinations simply forreader's convenience

### Suggested regimen as subsequent therapies for patients with advanced, metastatic or recurrent disease who have failed the frontline therapies

For patients experiencing treatment failure after frontline therapy, evidence advocates additional palliative systemic treatments to prolong survivals rather than supportive care alone [79]. Ramucirumab, an anti-VEGFR2 monoclonal antibody, has demonstrated efficacy in this clinical setting. The REGARD study showed that ramucirumab monotherapy provided superior survivals compared to placebo [80]. The RAINBOW study also demonstrated modest survival improvements when ramucirumab was combined with paclitaxel [81]. Additional chemotherapeutics including irinotecan, docetaxel, and paclitaxel represent reasonable alternatives for patients without prior exposure to these drugs. Furthermore, the TAGS study examined oral trifluridine-tipiracil (FTD-TPI) in heavily-pretreated cases, revealing additional OS improvements [82]. Likewise, the ATTRACTION-02 trial established survival benefits with nivolumab monotherapy in patients previously exposed to platinum and taxane combinations [83]. Collectively, these clinical trial outcomes have broadened the therapeutic landscape for second-line and subsequent treatments for patients with refractory disease.

Tumor agnostic approaches offered viable alternatives for patients with treatment-resistant disease. Targeted agents including entrectinib, larotrectinib, and repotrectinib have shown clinical benefit in tumors harboring NTRK gene fusions, while the combination of dabrafenib and trametinib proved effective for BRAF V600E mutations, and selpercatinib demonstrated activity against RET fusion-positive tumors, though these molecular alterations occurred extremely infrequent in the total population [84–86]. Additionally, while pembrolizumab monotherapy failed to show superior survival over paclitaxel in unselected populations, it demonstrated substantial efficacy in patients with MSI-H, dMMR or high TMB (≥ 10 mut/Mbps) tumors, which was selectively incorporated by some global guidelines [78, 87]. 

Moreover, for tumors exhibiting HER2 overexpression, the antibody-drug conjugate trastuzumab deruxtecan (T-Dxd) has shown substantial clinical activity in trastuzumab-refractory disease, as evidenced by the Western phase II DESTINY-Gastric02 (progression after trastuzumab exposure with retained HER2 positivity) and global phase III Gastric04 studies where it served as a subsequent-line option [88, 89]. The ongoing phase Ib/II DESTINY-Gastric03 trial is currently evaluating T-Dxd combined with immunochemotherapy in treatment-naïve cases, which could potentially change the treatment paradigm in the future [90]. 

The global guidelines have considerably demonstrated a broad alignment regarding therapeutic options beyond frontline therapy, endorsing either anti-VEGF therapy, chemotherapy, ICIs, or biomarker-driven targeted agents for managing refractory disease following initial treatment (Table 7). The CSCO guidelines constituted an exception by including some agents only available in China. Drawing upon this established evidence, the TWGCA guidelines advocated for single-agent taxanes or irinotecan, ramucirumab-paclitaxel combination, ICIs, and biomarker-driven treatments as appropriate strategies for second-line or subsequent therapies. (KQ 3-6)

**Table 7:** Suggested regimen as subsequent therapies for patients with advanced, metastatic or recurrent disease who have failed the frontline therapies

Guidelines	Second-line therapies		Third-line or above therapies		Note
TWGCA 2025 (KQ 3–6) (LoE: II; LoA: ***; GoR: C)	PTX + RAM Taxane IRI RAM	T-Dxd (HER2-positive disease) Biomarker-driven options^b^	FTD-TPI Nivo^c^		Clinical trial enrollment
JGCA 2025	PTX + RAM	*MSI-H*: Pembro^a^	*HER2-positive*: T-Dxd	*HER2-negative*: IRI FTD-TPI Nivo^c^	Clinical trial enrollment
KGCA 2024	PTX + RAM Taxane IRI RAM	*MSI-H/dMMR*: Pembro^a^	*HER2-positive*: T-Dxd	*HER2-negative*: IRI FTD-TPI Nivo^c^	Clinical trial enrollment
CSCO 2023	T (HER2-positive disease)^c^ PTX + RAM Taxane IRI (Grade I)	*MSI-H/dMMR*: Envafolimab Tislelizumab (Grade I)	*HER2-positive*: Disitamab vedotin Apatinib Nivo^c^ (Grade I)	*HER2-negative*: Apatinib Nivo^c^ (Grade I)	Clinical trial enrollment Suggest T if not exposed in frontline for HER2-positive disease
NCCN 2026 (version 1)	*Preferred*: PTX + RAM T-Dxd (HER2-positive disease) Taxane IRI (Category 1) FOLFIRI	*Others*: RAM (Category 1) IRI + P FOLFIRI + RAM IRI + RAM Docetaxel + IRI	*Under certain circumstances*: FTD-TPI (third-line or above) (Category 1) Biomarker-driven options^b^		Only stated “second-line or subsequent” instead of “second-” vs. “third-line” therapies Clinical trial enrollment
ESMO 2022, living guidelines 2024 (version 1.4)	PTX + RAM RAM Taxane IRI T-Dxd (HER2-positive disease)	*MSI-H/dMMR*: Pembro^a^	*HER2-positive*: T-Dxd^c^	*HER2-negative*: FTD-TPI Taxane IRI	Clinical trial enrollment
ESMO Pan-Asian adapted	Same above		Same above	*HER2-negative*: FTD-TPI Taxane IRI Nivo^c^	Clinical trial enrollment

a. if not previously exposed to immune checkpoint inhibitors

b. entrectinib, larotrectinib or repotrectinib for NTRK fusions; Pembro, Nivo + Ipi or dostarlimab for MSI-H/dMMR; Pembro for TMB ≥ 10 mut/Mbps; dabrafenib + trametinib for BRAF V600E mutation; selpercatinib for RET fusions-positive tumors

c. if not exposed in previous lines of treatment

HER2, human epidermal growth factor receptor-2; MSI/MMR, microsatellite instability/mismatch repair; TWGCA, Taiwan Gastric Cancer Association; JGCA, Japan Gastric Cancer Association; KGCA, Korean Gastric Cancer Association; CSCO, Chinese Society of Clinical Oncology; NCCN, National Comprehensive Cancer Network; ESMO, European Society for Medical Oncology;. Nivo, nivolumab; Ipi, ipilimumab; Pembro, pembrolizumab; PTX, paclitaxel; RAM, ramucirumab; IRI, irinotecan; T-Dxd, trastuzumab deruxtecan; FTD-TPI, trifluridine–tipiracil. LoE (II) publication(s) from at least one medium to low-quality RCT or one medium to low-quality meta-analysis. LoA (***) 60–79% of voting agreement in the expert panel. GoR (C) weakly recommended

### Role of intraperitoneal chemotherapy in patients with advanced, metastatic or recurrent disease

Multimodal strategies combining cytoreductive surgery with regional chemotherapy delivery, such as hyperthermic intraperitoneal chemotherapy (HIPEC) or pressurized intraperitoneal aerosol chemotherapy, aim to eradicate microscopic residual disease and achieve sustained tumor control [91]. However, these intraperitoneal treatments have yielded inconsistent outcomes due to uncertainties in patient selection, optimal regimens, and standardized protocols. The phase III GASTRIPEC-I trial assessed adding HIPEC to cytoreductive surgery after initial systemic therapy. While OS was not statistically significant, HIPEC demonstrated superiority in PFS and time to distant metastases [92]. On the other hand, the implication of normothermic intraperitoneal/systemic chemotherapy has been widely investigated in patients with peritoneal carcinomatosis using an implantable peritoneal infusion port. The preliminary report of phase III DRAGON-1 study, which compared intraperitoneal and intravenous paclitaxel plus S-1 versus intravenous paclitaxel plus S-1, demonstrated a superior survival benefit in patients with peritoneal metastasis [93]. Given limited robust evidence and various administration strategies, current recommendations still restrict the application to carefully selected candidates: those with limited peritoneal burden (peritoneal carcinomatosis index, PCI ≤ 10), disease confined to peritoneum, favorable systemic therapy responses, and eligibility for curative resection.

Intraperitoneal chemotherapy has not been adopted as standard care in the international guidelines (Table 8). The NCCN guidelines suggested HIPEC consideration for patients with peritoneum-restricted disease, sustained systemic therapy responses, and multidisciplinary evaluation. Other guidelines positioned HIPEC exclusively within research contexts. After comprehensive evidence review, the TWGCA panel determined HIPEC should remain highly selective, confined to patients with: (1) peritoneal-isolated metastases, (2) documented systemic treatment response, (3) minimal peritoneal burden (PCI ≤ 10), and (4) eligibility for complete curative resection. (KQ 2–5 and 3–7)

**Table 8:** Suggestions or definitions of intraperitoneal chemotherapy, reduction/palliative gastrectomy and oligo-metastatic disease

Guidelines	Intraperitoneal chemotherapy	Reduction/palliative gastrectomy (RG/PG)	Oligo-metastatic disease
TWGCA 2025 (KQ 2-5, 3-7 to 3-9)	Conditional for HIPEC^a^ (LoE: II; LoA: *****; GoR: B)	RG: Not recommended PG: Only for symptomatic relief (LoE: II; LoA: *; GoR: D)	No clear definitions Mentioned^b^ (1) solitary hepatic lesion, (2) limited LNs at No.16a2/b1 or (3) highly selected condition based on multidisciplinary work (LoE: V; LoA: *; GoR: D)
JGCA 2025	No recommendations	RG: Not recommended PG: Only for symptomatic relief	No clear definitions Mentioned^b^ (1) small number of LNs at No.16a2/b1, (2) solitary hepatic lesion
KGCA 2024	No recommendations Only for investigational purposes	RG: Not recommended PG: Only for symptomatic relief	No clear definitions Mentioned ≤ 3–5 lesions involving 1 or 2 organs Mentioned limited lesion(s) in the ovary or liver
CSCO 2023	No recommendations Conditional for ascites relief	No recommendations	1) LNs at No.16a2/b1 2) Solitary hepatic lesion^c^ 3) Ovary
NCCN 2026 (version 1)	Conditional for HIPEC: peritoneum-limited cases with multidisciplinary discussion	RG: Not recommended PG: Only for symptomatic relief	No clear definitions Mentioned “peritoneal carcinoma as only disease”^d^
ESMO 2022, living guidelines 2024 (version 1.4) and Pan-Asian adapted	No recommendations Only for investigational purposes	RG: Not recommended PG: Only for symptomatic relief	No clear definitions Conceptual framework: (1) limited burden, (2) response to chemotherapy, (3) in high-volume referral centers and (4) multidisciplinary work

a. peritoneum-only metastatic disease who have responses to systemic treatment, a PCI ≤ 10, and planned for cytoreductive surgery under a curative intent

b. plus the absence of other non-curable factors

c. defined as one ≤ 5 cm lesion which confines to single lobe of liver sparing nearby bile ductular or vascular structures

d. defined as PCI ≤ 10, peritoneal disease only in the absence of extra-peritoneal lesions, at least improved or stable disease after ≥ 3 months of systemic therapy and deemed possible for a complete cytoreduction

PCI, peritoneal carcinomatosis index; reduction gastrectomy, RG; palliative gastrectomy, PG; HIPEC, hyperthermic intraperitoneal chemotherapy; LN, lymph nodes; TWGCA, Taiwan Gastric Cancer Association; JGCA, Japan Gastric Cancer Association; KGCA, Korean Gastric Cancer Association; CSCO, Chinese Society of Clinical Oncology; NCCN, National Comprehensive Cancer Network; ESMO, European Society for Medical Oncology. LoE (II) publication(s) from at least one medium to low-quality RCT or one medium to low-quality meta-analysis; (V) publication(s) from reviews, expert opinions or commentaries. LoA (*) absence, (*****) 100% of voting agreement in the expert panel. GoR (B) moderately recommended; (D) insufficient to conclude

### Role of reduction/palliative gastrectomy in patients with advanced, metastatic or recurrent disease

When advanced or metastatic disease progresses to tumor bleeding, perforation, or viscus obstruction, immediate palliative surgical management focuses on symptom control and quality of life rather than cure. Separately, reduction/cytoreductive gastrectomy seeks maximum tumor removal combined with systemic therapy. The REGATTA trial investigated initial reduction gastrectomy with systemic treatment as frontline management in advanced, metastatic, or recurrent GC [94, 95]. Among non-emergent cases with only one non-curable factor, including liver (H1), peritoneum (P1), or distant para-aortic node (16a1/b2) involvement, upfront gastrectomy (D1 lymphadenectomy without metastatectomy) before chemotherapy showed no survival improvement. However, patients with node involvement confined to station 16a2/b1 were excluded.

International guidelines uniformly concluded that reduction gastrectomy should not be performed in advanced, metastatic, or recurrent disease given absent survival advantages. Palliative gastrectomy may be considered exclusively for urgent complications or symptomatic disease, without curative intent (Table 8). (KQ 3–8)

### Definition of oligo-metastatic lesion(s) in patients with advanced, metastatic or recurrent disease

Oligo-metastatic disease describes a clinical state characterized by limited metastatic burden within defined extent, representing a transitional status between locally confined (potentially curable with resection) and systemic (incurable) disease [96, 97]. The OligoMetastatic Esophagogastric Cancer (OMEC) initiative has proposed the entity as fewer than three metastatic lesions localized to a single organ site or isolated distant lymph node involvement [98, 99]. Establishing oligo-metastatic criteria serves to identify patients who might be candidates for conversion surgery [100]. The phase II AIO-FLOT3 trial suggested improved OS for patients who proceeded to surgery as compared to those not [101]. Subsequently, the retrospective CONVO-GC-01 analysis emphasized that achieving complete microscopic-free resection (R0) represents a critical determinant of outcomes in this oligo-metastatic population [102]. Conversely, the phase III RENAISSANCE (AIO-FLOT5) study did not confirm survival benefits when combining systemic therapy with metastasectomy [103]. These contradictory findings likely stem from elusive oligo-metastatic definitions and heterogeneous disease characteristics across organ sites and burden extents.

International guidelines have not achieved uniform agreement on oligo-metastatic disease, though various frameworks have been suggested. The JGCA guidelines characterize limited disease as involving ≤ 3 metastatic foci within liver or lung or nodal involvement at station 16a2/b1. The KGCA guidelines proposed limited lesion numbers in affected organs, identifying liver and ovarian sites as possible metastasectomy targets, albeit with low-strength recommendation. The CSCO guidelines specified single hepatic metastasis measuring under 5 cm, restricted to one hepatic lobe, without involvement of major vessels or biliary structures. In contrast, the NCCN guidelines focused on “peritoneum-only disease” suitable for peritoneal-directed and systemic therapies. The definitions for limited or oligo-metastatic disease are summarized in Table 8. After deliberating on this uncertainty, the TWGCA expert panel identified three potentially qualifying scenarios as oligo-metastatic: (1) isolated hepatic metastasis, (2) confined para-aortic nodal disease at station 16a2/b1, and (3) peritoneal cytology-positive disease only (CY1), each representing possible candidates for conversion therapy. Nevertheless, the suggestion received only modest consensus with lower-grade recommendation strength, acknowledging that rigorous definitions await validation through future prospective trials. (KQ 3-9)

## Conclusion

*The Taiwan Consensus and Management Guidelines for Gastric Cancer (2025)* represent a comprehensive and evidence-based framework developed through multidisciplinary collaboration and rigorous comparison with guidelines from Japan, South Korea, China, Europe, and US. This present comparative review reveals substantial global convergence on fundamental principles while highlighting important variations.

Across all guidelines, while a consensus exists in the absolute criteria for ER in early GC, nuanced differences appear in the extended indications, pre-procedural requirements and extension to surgically unfit patients. Likewise, a large divergence presents regarding perioperative therapy in locally advanced resectable disease, where Western guidelines prioritize chemotherapy or chemoimmunotherapy prior to surgery, yet some Asian-Pacific guidelines consider a relatively conservative approach. While international guidelines generally adopt IHC-based biomarkers, such as HER2, PD-L1, MSI/MMR and CLDN18.2, discrepancy is observed in prespecified cutoff thresholds, companion diagnostics and positioning of CGP. For metastatic or recurrent diseases, there is an alignment in addition of anti-HER2 therapy, anti-VEGF agents, ICIs, and biomarker-driven approaches. However, controversial domains persist in the definitions of oligo-metastatic disease and intraperitoneal chemotherapy.

This comparative review reinforces that optimal GC management requires integration of global evidence with regional contexts. As the therapeutic landscape continues to evolve, sustained international collaboration and periodic guideline updates will remain essential for advancing the quality of care.

## Supplementary Information

Below is the link to the electronic supplementary material.

Supplementary Material 1

## Data Availability

The data that support the descriptions of this review are available from open sources in the public domains.
