# Supplementary material for: Tackling similarities and differences in global practice guidelines for gastric cancer: a review on the latest Taiwan guidelines with Asia-Pacific, European and US guidelines
Source: Gastric Cancer. 2026 May 18;29(4):681–701. doi: 10.1007/s10120-026-01753-8 (PMC13315491; doi:10.1007/s10120-026-01753-8)
Supplement: Supplementary file 1 — Supplementary Material 1 [file 10120_2026_1753_MOESM1_ESM.docx]

Supplemental Materials

**Tackling similarities and differences in global practice guidelines for gastric cancer: a review on the latest Taiwan guidelines with Asia-Pacific, European and US guidelines**

Yan-Shen Shan, MD. PhD.

Department of Surgery, National Cheng Kung University Hospital, College of Medicine, National Cheng Kung University, Tainan, Taiwan

ysshan@mail.ncku.edu.tw

Cheng-Hsing Campus, No.1, University Road, Tainan, Taiwan

Tel: +886-6-235-3535 ext. 5015

**Supplemental 1. The composition of taskforce for *Taiwan consensus and management guidelines for gastric cancer (2025)***

A multidisciplinary consortium was composed of 36 specialists from 17 Taiwanese medical institutes collaborated to develop the present guidelines. The panel composition reflected diverse expertise across five professional societies, including the Taiwan Oncology Society (TOS), Taiwan Society for Immunotherapy of Cancer (TSITC), Gastroenterological Society of Taiwan (GEST), Taiwan Surgical Society of Gastroenterology (TSSG), and Taiwan Gastric Cancer Association (TWGCA), each contributing panelists with demonstrated proficiency in GC management. Panelist expertise encompassed four key domains: gastrointestinal oncology (15 medical oncologists), surgical intervention (14 specialists in both open and minimally invasive gastric procedures), endoscopic therapy (6 gastroenterologists and endoscopists), and molecular research (2 pathologists focused on biomarker and translational investigation). This diverse representation ensured comprehensive coverage of clinical and research perspectives.

**Supplemental 2. Key questions (KQ) and recommendation statements (RS) from the *Taiwan Consensus and Management Guidelines for Gastric Cancer (2025)***

***KQ 1-1***: ***What are the recommended methods for early GC screening?***

In formulating recommendations for GC screening in Taiwan, the expert panel reviewed the international GC prevention guidelines, along with supporting evidence from regional programs in Japan and South Korea. Emphasis was placed on identifying a screening strategy that could efficiently target high-risk individuals while making optimal use of existing healthcare resources. Based on the reviewed evidence and expert discussion, a moderate level of agreement (71%) was reached among panelists to suggest that individuals aged 45–74 years with a positive *Helicobacter pylori (HP)* test (via stool antigen, urea breath test, or serum/urine antibody) undergo a one-time EGD screening, followed by risk-based surveillance using image-enhanced endoscopy (IEE). This targeted approach enhances early detection and GC prevention while optimizing healthcare resource utilization in a locally applicable manner.

According to the recently published *Taipei Global Consensus II on Screening and Eradication of Hp for GC Prevention in Gut*, surveillance is recommended for patients with advanced atrophic gastritis or intestinal metaplasia—such as those with Operative Link on Gastritis Assessment (OLGA) or Operative Link on Gastric Intestinal Metaplasia Assessment (OLGIM) stage III–IV, open-type atrophy based on the Kimura–Takemoto classification, or abnormal serum pepsinogen levels. In addition, patients who have undergone endoscopic resection or gastrectomy should also undergo regular surveillance due to the high risk of developing metachronous gastric cancer.(1)

**RS 1-1: Individuals aged 45–74 years with a positive *Hp* test (via stool antigen, urea breath test, or serum/urine antibody) should undergo a one-time EGD, followed by risk-based image-enhanced endoscopic (IEE) surveillance. (LoE: III; LoA: **; GoR: B)**

***KQ 1-2:*** ***Are stool- or blood-based assays recommended for pre-selecting the high-risk population?***

Both stool- and blood-based assays received a weak consensus from the expert panel (each with 46% agreement) for identifying individuals at increased risk of GC due to *Hp* infection. The stool antigen test is valuable for detecting current active infection, thereby guiding timely eradication therapy and initial endoscopic evaluation. In contrast, the serum *Hp* IgG antibody test reflects antecedent *Hp* infection and is useful for identifying individuals who may still carry GC risk even after *Hp* eradication.

These assays have been incorporated into large-scale screening and triage programs in Taiwan and Japan, where serum pepsinogen and *Hp* IgG are commonly used to stratify GC risk and determine surveillance intervals. While the stool antigen test offers high specificity for active infection, the serum antibody test is advantageous for identifying those with prior exposure who may still benefit from monitoring due to mucosal atrophy or residual cancer risk. Urea breath test is not considered cost-effective for mass screening. Collectively, these non-invasive assays are essential tools for pre-selecting high-risk individuals before proceeding to more resource-intensive endoscopic evaluations.

**RS 1-2: Use of stool antigen testing (to detect current *Hp* infection) and/or serum *Hp* IgG antibody testing (to detect past or ever Hp infection) is recommended for pre-selecting individuals at increased risk for GC. (LoE: III; LoA: *; GoR: D)**

***KQ 1-3:*** ***What is/are the required pre-procedure exam(s) for patients planning to receive endoscopic mucosal resection (EMR) or endoscopic submucosal dissection (ESD) for early GC?***

Contrast-enhanced computed tomography (CT), especially multidetector CT with multi-planar reconstructions, improves detection of loco-regional disease and distant metastases. Its accuracy in distinguishing T1 from advanced GC ranges from 64% to 95%, but is limited for T1a vs. T1b.(2) (3) (4) Endoscopic ultrasonography (EUS), particularly with high-frequency probes, offers superior depth assessment but is operator-dependent. A Cochrane review reported EUS pooled sensitivity and specificity of 86% and 90% for differentiating T1/T2 from T3/T4, and 87% and 75% for T1a vs. T1b. A Cochrane review reported pooled sensitivity and specificity of EUS for distinguishing superficial (T1/T2) vs. from advanced (T3/T4) GC at 86% and 90%, respectively.(5) NCCN also recommended EUS when early-stage disease is suspected.(6)

Unlike Western guidelines, Japanese guidelines prioritize endoscopic assessment for early GC staging. Conventional white-light endoscopy aids depth estimation, while magnifying endoscopy combined with narrow-band imaging (ME-NBI) allows experienced endoscopists to assess invasion depth and spread through pattern analysis. (7, 8) EUS is a helpful adjunct when endoscopic assessment of invasion depth is unclear. For nodal staging, CT shows 49.3%–79.5% accuracy, while EUS varies from 30%–90% and is operator-dependent(9, 10) A large study reported EUS accuracy of 66.7%(11), with operator-dependent, limited assessment of distant nodes due to restricted depth and field of view.(12)

The expert panel reviewed NCCN, ESMO, and Asian guidelines, as well as recent studies on imaging accuracy in GC staging. Recognizing the lack of universally superior modality and variability in institutional resources, 64% of panelists agreed that contrast-enhanced CT as mandatory. EUS or ME-NBI should be added as adjuncts, particularly for patients being considered for endoscopic resection, to enhance depth and nodal assessment.

**RS 1-3: CT is mandatory, ME-NBI or EUS should be added to assess invasion depth and lesion extent. (LoE: IV; LoA: ***; GoR: C)**

***KQ 1-4:*** ***If the pre-procedural exam for EMR/ESD suggests controversial perigastric lymph node metastasis, what is the recommended management?***

During panel discussion, only limited panelists (21%) supported using ME-NBI to further evaluate the primary lesion and assess suitability for endoscopic resection. Evidence shows that depth of invasion, tumor size, and histologic type are key risk factors for nodal metastasis. Japanese guidelines consider endoscopic evaluation effective for invasion depth, with minimal nodal risk when absolute resection criteria are met.(13) However, in situations where imaging raises concern for possible lymph node metastasis—despite the absence of definitive confirmation—71% of panel members agreed proceeding with surgery as a precautionary measure.

**RS 1-4: If lymph node metastasis is suspected based on imaging or endoscopic assessment, surgery is recommended for caution’s sake. (LoE: V; LoA: ***; GoR: D)**

***KQ 1-5:*** ***Could EMR/ESD be applied to patients with poorly differentiated or signet-ring cell histology?***

ESD is a suitable therapeutic option for early GC patients with poorly differentiated adenocarcinoma or signet-ring cell histology within clearly defined extended criteria. The balance between high technical efficacy, promising long-term survival, and reduced morbidity highlights its clinical value. Given the elevated risk of recurrence, meticulous patient selection, procedural precision, and stringent post-procedure surveillance remain essential.

In the panel discussion, only 71% agreement was achieved. Considering the risks, still part of the panelists suggested a stringent criterion on the operator’s experience and pre-procedural thorough image evaluation. We have also reviewed data from one university hospital in Taiwan, showing less procedural time, less R0 resection but comparable survival. Therefore, the expert panel concluded a conditional endorsement for extending the indication of ESD in patients with poorly differentiated or signet-ring cell histology.

**RS 1-5: ESD can be applied to poorly differentiated or signet-ring cell histology if the lesion is limited to mucosa layer, size less than 20mm without ulcerations, negative nodal or distal metastases by CT. (LoE: IV; LoA: ***; GoR: C)**

***KQ 1-6:*** ***Could EMR or ESD be applied to patients with clinical T1b lesion?***

Risk stratification significantly influences the incidence of lymph node metastasis. In patients meeting the expanded submucosal invasion criteria—defined as submucosal invasion <500 μm, differentiated-type histology, no lymphovascular invasion, and tumor size ≤3 cm—the metastasis incidence was 2.6%.(14)

In a meta-analysis, patients who met the expanded submucosal invasion criteria had a significantly higher incidence of lymph node metastasis compared to those who met the absolute criteria (8/315 [2.5%] vs. 3/850 [0.35%]). The relative risk of lymph node metastasis was 6.30 (95% CI: 1.79–22.16; P = .004) favoring the absolute criteria in subgroup analysis.(15) These findings indicate that while ESD is technically feasible for cT1b cases, its limited curative potential and higher nodal involvement risk warrant caution. It should be reserved for patients unfit or unwilling to undergo surgery, which remains the preferred option for superior oncologic outcomes.

In formulating this recommendation, the expert panel all agree with the higher nodal metastases risk of T1b lesion. With 93% agreement, the panel recommends surgery as the preferred treatment. If surgery is not performed due to comorbidities or patient refusal, strict eligibility criteria should be applied, and close surveillance is essential.

**RS 1-6: ESD can be applied to patients with clinical T1b lesion if the lesion is limited to superficial submucosa (< 500um), size less than 30mm, negative nodal or distal metastases by CT scan, and negative for lymphovascular invasion in biopsied specimens. (LoE: IV; LoA: ****; GoR: C)**

***KQ 1-7:*** ***When the gastric lesion is resected in a piecemeal manner or the margins inadequate for a clear evaluation, could ESD be repeated?***

Management of gastric lesions with piecemeal resection, non-evaluable (Rx), or R1 resection remains controversial due to variable oncologic risk and heterogeneous evidence. Owing the absence of standardized definitions, the panel favored surgery as the primary recommendation. Nonetheless, in selected patients with isolated horizontal margin positivity, additional endoscopic therapy may still be appropriate. In our panel, 50% opposed repeating ESD after piecemeal resection or unclear margins, 29% supported it only under a prespecified low-risk condition, 14% reserved it for non-surgical candidates, and 7% stressed multidisciplinary input. The final consensus favored surgery but allowed repeat ESD in select low-risk cases at expert centers.

**RS 1-7: Surgery is generally recommended for patients with piecemeal resection or unclear margins after ESD. However, repeat ESD may be considered in selected cases with low-risk features if performed at expert centers. (LoE: II; LoA: **; GoR: D)**

***KQ 1-8: Could EMR/ESD be recommended for early GC treatment over surgery for unfit patients?***

Emerging data has supported ESD as a potentially curative alternative for medically inoperable early GC patients, especially when lesions meet or approximate the expanded criteria and are followed by proper surveillance. Although not yet standard in guidelines, evidence affirms its adequacy in highly select cases.

During consensus discussions, three initial questions were consolidated into a single key question: Can EMR/ESD be recommended over surgery in unfit patients? This reflects a standard shift from recognition of fitness rather than chronological age, ultimately endorsing ESD/EMR for unfit patients when endoscopic criteria are met. Collectively, these studies support the consideration of ESD as a potentially curative approach in medically inoperable patients with early GC, particularly when the lesions fulfill or approximate expanded indications and appropriate post-procedural surveillance is ensured. While existing clinical guidelines have yet to formally incorporate this approach into standard recommendations for unfit patients, growing evidence supports its oncological validity in appropriately selected populations.

**RS 1-8: EMR/ESD is recommended as a treatment option for early GC in patients unfit for surgery due to age, comorbidity, or frailty, provided the lesion fulfills standard criteria for endoscopic curability. (LoE: IV; LoA: ****; GoR: C)**

***KQ 2-1:*** ***What is/are the recommended exam(s) for selecting patients with locally advanced GC planning to receive perioperative therapies?***

Considering the cost-effectiveness of selecting appropriate candidates for perioperative therapies, CT imaging remains the gold standard for pretreatment evaluation. EUS, upper gastrointestinal tract series imaging (UGIS) and positron emission tomography (PET)/CT imaging as well as diagnostic laparoscopy/washing cytology provide additional complementary information. In summary, the expert panel concluded that CT imaging is the mandatory pretreatment exam for patients with locally advanced disease planning to receive perioperative therapies. EUS, UGIS, PET/CT and diagnostic laparoscopy/washing cytology are optional exams which provide relevant information in addition to CT imaging.

**RS 2-1: For patients planning to receive perioperative therapies, contrast-enhanced CT imaging is the mandatory exam. EUS, UGIS, PET/CT imaging and diagnostic laparoscopy/washing cytology are optional pretreatment exams providing additional relevant information. (LoE: II; LoA: ****; GoR: B)**

***KQ 2-2:*** ***What is the recommended field of imaging for patients with locally advanced GC planning to receive perioperative therapies?***

The primary purpose for CT imaging prior to treatment is accurate TNM staging, particularly the detection of distant metastases (M category), which is essential for treatment planning. Although CT does not offer perfect resolution, imaging from the chest to the pelvis effectively detects metastatic lesions in the liver, peritoneum, lungs, and ovaries.(16) In addition, the presence of pleural, pericardial, or peritoneal effusions provides clinically relevant information for treatment decision-making. Limiting the scan field may lead to underestimation of disease extent in locally advanced GC, thereby compromising optimal treatment decisions.

In summary, the expert panel concluded that a comprehensive CT imaging, covering chest, abdomen to pelvis, is required as the pretreatment examination for patients with locally advanced GC to ensure optimal treatment planning. This recommendation is supported by a moderate level of evidence and panel agreement.

**RS 2-2: For patients with locally advanced disease planning to receive perioperative therapies, a contrast-enhanced CT imaging covering chest, abdomen and pelvis is recommended prior to treatment initiation. (LoE: II; LoA: ***; GoR: B)**

***KQ 2-3:*** ***How do we select patients with locally advanced GC for perioperative therapies?***

Among all Western perioperative chemotherapy protocols, the FLOT regimen (fluorouracil, leucovorin, oxaliplatin, and docetaxel) is considered the optimal choice. The AIO-FLOT4 trial demonstrated the efficacy and survival benefits of FLOT as compared with anthracycline-platinum-fluoropyrimidine triplet in patients with ≥cT2N(+) locally advanced GC.(17) Major Western guidelines, including NCCN and ESMO, have recognized that FLOT is the standard of care for patients planning to receive perioperative therapy.

The phase III Korean PRODIGY trial evaluated preoperative chemotherapy using docetaxel, oxaliplatin, and S-1 (DOS), followed by surgical resection and postoperative S-1 therapy in patients with cT2-3N(+) or cT4 disease. The results indicated a significant favorable OS and improved surgical outcomes.(18) The RESOLVE study, which was carried out in China, investigated perioperative S-1 and oxaliplatin (SOX) in patients with cT4aN(+) or cT4b disease. Perioperative SOX treatment demonstrated meaningful disease-free and overall survival improvements compared to adjuvant CAPOX.(19)

The phase III KEYNOTE-585 trial examined the efficacy of pembrolizumab plus chemotherapy versus chemotherapy alone but failed to meet its primary endpoint, despite that the pathological complete response rate had significantly increased.(20) On the other hand, the phase III MATTERHORN trial, which incorporated durvalumab plus FLOT, has been proven to significantly improve survivals and increase optimal pathological regression as compared to chemotherapy alone, which is expected to change the treatment paradigm.(21)

In summary, the expert panel concluded that patients who are fit for systemic treatments with either cT2-3 plus N(+) M0 or cT4 plus any N M0 disease are appropriate candidates to receive perioperative therapies with highest level of evidence and a high level of voting agreement.

**RS 2-3: Patients who are eligible for systemic treatments are recommended candidates for perioperative therapies if they have either cT2-3 plus N-positive M0 or cT4 plus any N M0 disease. (LoE: I; LoA: ****; GoR: B)**

***KQ 2-4:*** ***What are the ideal therapeutic regimens for perioperative therapies in locally advanced GC?***

Given the existing evidence and geographical variations between Western and Asian practice guidelines, the expert panel concluded platinum-fluoropyrimidine-taxane triple chemotherapy, or its equivalent combinations with/without an immune checkpoint inhibitor (ICI) are the backbones of perioperative therapies. Doublet chemotherapy is an alternative for patients who are ineligible for triplet regimens. These recommendations do not preclude the incorporation of novel agents. The conclusion was supported by the highest level of evidence and strong voting consensus.

**RS 2-4: Platinum-fluoropyrimidine-taxane triple chemotherapy or its equivalent combinations are the backbones of perioperative therapies with/without an immune checkpoint inhibitor. Doublet chemotherapy is an alternative when patient is not eligible to receive triplet combinations. (LoE: I; LoA: ****; GoR: B)**

***KQ 2-5:*** ***Is prophylactic hyperthermic intraperitoneal chemotherapy (HIPEC) recommended for patients with locally advanced M0 GC?***

Introducing HIPEC in patients absent for clinical evidence of peritoneal metastasis, or “prophylactic HIPEC”, remains controversial with inconsistent results. Selective studies have indicated that patients with a high risk for occult peritoneal metastasis, such as cT4 disease or bulky LN involvement, may benefit from prophylactic HIPEC during curative gastrectomy in terms of locoregional control and recurrence-free survival.(22-24) However, an exclusion of patients with frank peritoneal carcinomatosis is essential to avoid the unnecessary procedure in patients with M1 disease, whose treatment goal should be a maximal cytoreduction and disease palliation.

In summary, given the limited but emerging evidence and the need for improved locoregional peritoneal control, the expert panel concluded that prophylactic HIPEC may be considered only for patients with cT4 disease or bulky nodal metastases, in the absence of peritoneal carcinomatosis on imaging or laparoscopy. This recommendation is based on moderate-level evidence and received a low level of voting agreement.

**RS 2-5: Prophylactic HIPEC is only indicated when patients have either cT4 disease, or bulky lymph node metastasis in the absence of peritoneal carcinomatosis on imaging or laparoscopic inspections. (LoE: III; LoA: **; GoR: D)**

***KQ 2-6:*** ***What are the recommended postoperative adjuvant therapies for patients with locally advanced disease?***

Given the existing evidence and geographical variations between Western and Asian practice guidelines, the expert panel concluded that adjuvant S-1 or CAPOX is recommended for pStage II disease, while S-1-based doublets, CAPOX, or FOLFOX are recommended for pStage III disease. The ideal initiation time for postoperative adjuvant therapy should range from 4 to 12 weeks in the postoperative period given that no immediate surgery-related complications are present. The recommendation is supported by a high level of evidence and a moderate level of voting agreement.

**RS 2-6: Adjuvant S-1 or CAPOX is indicated for patients with pStage II disease after surgery. Adjuvant S-1-based doublets, CAPOX or FOLFOX is indicated for patients with pStage III disease. (LoE: II; LoA: ***; GoR: B)**

***KQ 3-1:*** ***For patients with advanced, metastatic or recurrent disease, what are the recommended biomarker assays prior to systemic treatment?***

Immunohistochemical staining (IHC) and sequencing/NGS-based assays provide essential biomarker information to guide treatment selection. Given their shorter turnaround time and lower resource requirements, IHC assays remain the most cost-effective and practical option for treatment-naïve cases, while molecular testing can be reserved for cases with negative or inconclusive IHC results. Biomarkers such as HER2, immune checkpoints (e.g., PD-1/PD-L1, CTLA-4), and CLDN18.2 have been associated with significant therapeutic and survival benefits in treatment-naïve patients with advanced, metastatic or recurrent GC.(25-27) Various large phase III RCTs and meta-analyses have indicated the superiority of these combinations as compared with conventional chemotherapies. Therefore, to maximize the treatment efficacy of frontline therapies, the companion IHC-based biomarker assays should be conducted as a fundamental for therapeutic decision-making.

In light of the published results and consideration for local guidance and limitations, the expert panel concluded a recommendation that IHC-based assays, such as HER2, PD-L1, MMR and CLDN18.2, as essential assessments prior to systemic treatment for patients with advanced, metastatic or recurrent disease, supported by the highest level of evidence and voting agreement. However, such recommendations did not preclude the possible addition of other novel experimental biomarkers which are under development or validation in clinical trials.

**RS 3-1: HER2, PD-L1, MMR and CLDN18.2 IHC-based assessments are recommended biomarker assays prior to systemic treatment for patients with advanced, metastatic or recurrent disease. (LoE: I; LoA: *****; GoR: A)**

***KQ 3-2: For patients with advanced, metastatic or recurrent disease, is tumor-based microsatellite stability testing (MSI) routinely required for these patients?***

MSI testing, performed via PCR or NGS-based methods, generally requires a longer turnaround time than IHC-based assays, potentially delaying the initiation of systemic treatment. In addition, MSI-H population is expected to largely overlap with dMMR cases and leaves only a minor possibility of a positive yield. Therefore, when the resources limitation and cost/time-effectiveness are contemplated, the expert panel concluded that tumor MSI assay should be regarded as a companion assay for tumor agnostic purposes and not routinely required in all patients with advanced, recurrent or metastatic disease, when IHC-based dMMR assay is available in a timely fashion.

**RS 3-2: Tumor MSI testing, either via PCR or NGS-based method, is an optional companion assay for tumor agnostic purposes and not routinely required prior to systemic treatment. (LoE: II; LoA: ****; GoR: B)**

***KQ 3-3:*** ***How do we organize the recommended biomarker assays in patients with advanced, metastatic or recurrent disease?***

Given that conventional IHC-based assays can yield results efficiently when adequate tumor specimens are available, it is preferable to assess all relevant IHC biomarkers simultaneously to maximize diagnostic yield. Sequential testing inevitably leads to delays in interpretation and unnecessary waiting. Several reports indicated a desired turnaround interval of 7 to 10 days in a simultaneous IHC biomarker panel and a requirement for another longer period for additional biopsies when the first biopsy not conclusive or adequate.(28, 29) Therefore, the expert panel recommended simultaneous testing for currently available and validated IHC biomarkers, including HER2, PD-L1, MMR and CLDN18.2 at the time of initial diagnosis or systemic treatment planning in patients with advanced, metastatic, or recurrent disease. The recommendation statement was derived from a moderate level of evidence and highest level of voting agreement. However, such recommendations did not preclude the possible addition of other novel experimental biomarkers which are under development or validation in clinical trials.

**RS3-3: When tumor specimen is adequate, simultaneous testing for HER2, PD-L1, MMR and CLDN18.2 is recommended in patients with advanced, metastatic or recurrent disease. (LoE: II; LoA: *****; GoR: A)**

***KQ 3-4:*** ***Is tumor- or plasma-based panelized sequencing assay recommended for patients with advanced, metastatic or recurrent disease?***

Tumor agnostic targets have been proposed to correlate with therapeutic values in GC, such as TMB, BRAF mutations, NTRK or RET fusions, FGFR2, EGFR, or MET amplifications.(30) However, such population still constitutes a relatively small proportion of patients with its detection rate significantly lower than that of the IHC-based biomarkers. In addition, the turnaround time, testing availability and reproducibility of panelized genomic sequencing raise the issue of clinical utility when cost-effectiveness and timeliness are considered. Therefore, the expert panel concluded that either tumor- or plasma-based genomic sequencing could be recommended when IHC-based assays reveal a negative result in patients with advanced, metastatic, or recurrent disease with a low level of evidence and low voting agreement.

**RS3-4: Panelized sequencing assay, either by tumor- or plasma-based testing, is recommended when IHC-based assessments reveal a negative result in patients with advanced, metastatic or recurrent disease. (LoE: V; LoA: **; GoR: C)**

***KQ 3-5:*** ***What are the recommended frontline therapeutic combinations for patients with advanced, metastatic or recurrent disease?***

Platinum-fluoropyrimidine (Plt-F) doublet chemotherapy remains the cornerstone of systemic therapy. In HER2-positive patients with advanced GC, phase III trastuzumab for Gastric Cancer (ToGA) trial demonstrated that adding trastuzumab (Tmab), an anti-HER2 monoclonal antibody, to Plt-F-based regimens significantly improved OS (median survival 13.8 compared to 11.1 months; HR: 0.74; 95% CI: 0.60–0.91; P=0.0046) and the advantage was particularly prominent in tumors with strong HER2 overexpression.(31)

In addition, the randomized phase III KEYNOTE-811 trial further evaluated the efficacy of adding pembrolizumab to Tmab plus standard Plt-F as compared with the TOGA regimen. Interim analysis reported a significantly improved PFS and a meaningful elongation of OS in treatment-naïve HER2-overexpressed advanced GC. The survival and response advantages were especially prominent in tumors with PD-L1 CPS ≥1 (by pharmDx Dako 22C3). Therefore, a combination of pembrolizumab, Tmab and Plt-F has been recommended in patients with advanced GC whose PD-L1 CPS ≥1.(32, 33)

The KEYNOTE-859 trial showed meaningful OS improvements in treatment-naïve patients when pembrolizumab was added to Plt-F doublet.(34) The survival benefits were observed across all subgroups of patients but particularly prominent in patients with PD-L1 CPS ≥10. Another PD-1 inhibitor nivolumab also pioneered the successful frontline combination plus chemotherapy in patients with HER2-negative advanced unresectable GC. The results from CheckMate-649 study clearly demonstrated that adding nivolumab to capecitabine or 5-FU/oxaliplatin prolonged OS as compared to chemotherapy alone.(35) Despite that the survival benefits were observed across all patients regardless of the PD-L1 expression, patients with PD-L1 CPS ≥5 still derived the best survival outcomes (by pharmDx Dako 28-8). However, another East Asian-limited phase III trial, ATTRACTION-4, also evaluated the first line nivolumab plus chemotherapy in the identical population. Although the study failed to reveal similar significant OS differences in other trials, it showed meaningful improvements in PFS with addition of nivolumab.(36)

Noticeably, another ICI tislelizumab had been investigated in similar conditions. The global phase III RATIONALE-305 trial compared tislelizumab plus chemotherapy with chemotherapy alone in patients with untreated HER2-negative advanced disease. The study achieved its primary goals, revealing significant survival improvements among all enrolled patients.(37) Similarly, more pronounced benefits were observed in patients with high PD-L1 expressions, as assessed using the Ventana SP263 assay as the companion diagnostic. The results led to the US Food and Drug Administration (US-FDA) approval of tislelizumab in combination with chemotherapy in unresectable advanced treatment-naïve HER2-negative GC.

The phase III trials SPOTLIGHT and GLOW have established the efficacy of combining zolbetuximab with either FOLFOX or CAPOX as initial treatment for patients with CLDN18.2-positive, HER2-negative advanced disease.(38, 39)

Although tumors with MSI-H or dMMR represent a small population, comprising around 5% of all cases, the therapeutic potential of reinvigorating antitumor immune response with ICIs have been proposed. Post-hoc analyses from large clinical studies including CheckMate-649, KEYNOTE-062, and KEYNOTE-859 have uniformly revealed substantial clinical advantages when anti-PD-1 ICIs were incorporated with chemotherapy for initial frontline treatment in patients with advanced unresectable MSI-H/dMMR GC.

Considering the relevant results from large RCTs and meta-analyses, the expert panel concluded that the addition of ICIs or targeted agents to the Plt-F doublet is recommended based on the selection of a corresponding biomarker assay, with the highest level of evidence and voting agreement. However, in the real-world scenario, treatment considerations for elderly or frail patients, especially those with poor performance status, or comorbidities who are often underrepresented in clinical trials, should be carefully weighed for intensive systemic treatment protocols and adjustments should be applied tailored to the clinical condition.

**RS 3-5: Platinum-fluoropyrimidine doublet is the backbone of frontline systemic combinations, with addition of ICIs or targeted agents based on biomarker assays. (LoE: I; LoA: *****; GoR: A)**

***KQ 3-6:*** ***What are the recommended subsequent therapeutic combinations for patients with advanced, metastatic or recurrent disease who have failed the frontline therapies?***

Ramucirumab, a monoclonal antibody targeting vascular endothelial growth factor receptor 2 (VEGFR2), has been investigated in the second-line treatment for patients who failed on Plt-F chemotherapy. In the REGARD trial, ramucirumab monotherapy significantly improved OS and PFS as compared with placebo. Furthermore, the phase III RAINBOW trial assessed the combination of paclitaxel and ramucirumab, confirming a marginally prolonged OS as compared with paclitaxel alone.

Tumor agnostic therapies selected by panelized genomic sequencing remain alternative options when conventional treatments have failed.(40-42) In addition, although pembrolizumab monotherapy did not demonstrate superior survival advantages when compared with paclitaxel in the unselected population, it demonstrated substantial efficacy in treating patients whose tumor were designated to MSI-H, dMMR or high TMB (≥10 mutations/megabase) status.(43, 44)

Trastuzumab deruxtecan (T-Dxd), an antibody-drug conjugate that targets HER2 with a topoisomerase inhibitor payload, was approved as a third or later line of treatment for HER2-positive GC who have progressed under Tmab-containing therapy.(45) The phase III DESTINY-Gastric04 trial also confirmed the therapeutic efficacy of T-Dxd as compared with paclitaxel plus ramucirumab in patients with HER2-positive GC who have failed Tmab-containing therapy.(46)

The established evidence supports the expert panel to conclude that taxane or irinotecan monotherapy, paclitaxel plus ramucirumab or tumor-agnostic therapies are recommended second-line treatments. Enrolling patients into interventional clinical trials is an alternative option, with a high level of evidence and moderate level of voting agreement.

**RS 3-6: Taxane or irinotecan monotherapy, paclitaxel plus ramucirumab or tumor agnostic therapies are recommended second-line treatments. Enrolling patients into interventional clinical trials is an alternative option. (LoE: II; LoA: ***; GoR: C)**

***KQ 3-7:*** ***Is HIPEC recommended for patients with advanced, metastatic or recurrent disease?***

The addition of HIPEC improved disease control, PFS and OS in selected population given an optimal cytoreduction and adequate systemic treatment, even though peritoneal carcinomatosis index (PCI) remained static after the intervention.(47, 48) The GASTRIPEC-I phase III study examined cytoreductive surgery with or without HIPEC following initial chemotherapy. Despite that OS differences were not statistically significant, PFS and distant metastasis-free intervals favored HIPEC inclusion.(49) A general consensus indicated that HIPEC should be reserved for patients with low PCI (PCI ≤10), peritoneal-only metastasis, sustained responses to systemic treatment and planning for cytoreductive surgery under a curative intent.

The expert panel concluded that HIPEC could be recommended to patients with advanced GC and peritoneal disease under strict circumstances. It should be reserved for patients with peritoneal-only metastasis, who have responses to systemic treatment, a limited extent of peritoneal involvement (PCI ≤10) and planning to receive optimal cytoreductive surgery under a curative intent, with a moderate level of evidence and strong voting agreement.

**RS 3-7: HIPEC is recommended in patients with peritoneum-only metastatic disease** **who have responses to systemic treatment, a PCI ≤10, and planned for cytoreductive surgery under a curative intent. (LoE: II; LoA:*****; GoR: B)**

***KQ 3-8:*** ***Is palliative gastrectomy recommended for patients with advanced, metastatic or recurrent disease?***

The REGATTA trial was the first and the only randomized controlled trial evaluating the survival benefit of reduction surgery before chemotherapy as first-line treatment of advanced gastric cancer.(50) For asymptomatic advanced gastric cancer patients with one non-curative factor conﬁned to either the liver (H1), peritoneum (P1), or para-aortic lymph nodes (16a1/b2), upfront gastrectomy (D1 gastrectomy without metastatectomy) followed by chemotherapy did not show any survival beneﬁt compared with chemotherapy alone (HR: 1.08; 95% CI: 0.74–1.58; P=0.66). Reduction gastrectomy is not recommended in asymptomatic patients with metastatic gastric cancer. In this study, paraaortic lymph nodes metastasis does not include the station 16a2/b1, which is located inside the field of extended paraaortic lymph nodes dissection.

For the patients with oligo-metastasis, the role of radical D2 gastrectomy with metastasectomy followed by systemic therapy and conversion surgery remain controversial. Therefore, the expert panel concluded that palliative or cytoreductive gastrectomy is not routinely recommended and should be considered on a case-by-case basis within a multidisciplinary team discussion.

**RS 3-8: Palliative or cytoreductive gastrectomy is not routinely recommended and should be discussed under a multidisciplinary panel on a case-by-case basis. (LoE: II; LoA:*; GoR: D)**

***KQ 3-9:*** ***How do we define limited or oligo-metastatic disease which could potentially be converted to curative surgery?***

In a large-scale international retrospective cohort study, the CONVO-GC-1 study. The median OS for all resected patients was 36.7 months, with OS for R0, R1, and R2 resections being 56.6, 25.8, and 21.7 months, respectively.(51) In the phase II AIO-FLOT 3 study, the median OS was 31.3 months (95% CI: 18.9-upper level not achieved) for patients who proceeded to surgery and 15.9 months for the other patients.(52) However, the phase III RENAISSANCE (AIO-FLOT5) trial demonstrated no survival benefit from surgical resection following systemic therapy.(53) High surgical mortality (9%) and morbidity (60%) in the surgery group were noted. In the subgroup analysis, patients with retroperitoneal lymph nodes metastases only seemed to benefit most from the surgical approach.

In an expert consensus meeting at KINGCA WEEK 2024, most experts agreed that conversion therapy provides a survival benefit for selected patients who respond to systemic therapy and undergo R0 resection.(54) Patients with limited metastases were deemed good candidates. The panel proposed a definition of limited metastasis with over 80% consensus, including solitary liver metastasis, paraaortic lymph nodes metastasis at station 16a2/16b1, and isolated positive peritoneal cytology only, which were classified as Yoshida classification category 1 (technically resectable). Other definitions with less consensus included not greater than 3 lesions in unilobar or bilobar liver metastasis, unilateral or bilateral ovarian metastasis, unilateral adrenal gland metastasis, limited peritoneal metastasis, and paraaortic lymph nodes station 16a1/16b2 metastasis.

The optimal surgical timing was based on the best response to systemic therapy. The regimen was recommended to be individualized and the duration to be at least 6 months. A minimally invasive approach and D2 lymph node dissection were considered for surgery. However, resection for metastases with a complete clinical response after systemic therapy was not advocated. Large-scale randomized-controlled trials for further evidence is necessary. Therefore, with a general paucity of evidence-supported prospective results, the expert panel concluded that the definition for limited or oligo-metastatic disease which could be converted to curative surgery remains elusive and should not be performed as routine practice.

**RS 3-9: Limited or oligo-metastatic disease which could be converted to curative surgery remains inconsistently defined and should not be regarded as routine practice. (LoE: V; LoA:*; GoR: D)**

**Supplemental 3. Evidence-based approaches and grading for recommendations**

During each consensus meeting, panelists received comprehensive materials, including evidence syntheses from published literature, current practice patterns in Taiwan, and applicable regulatory frameworks. Panelists provided anonymous ratings on proposed recommendations using a five-point scale, where 1 indicated strong disagreement and 5 with a strong agreement. A recommendation achieved consensus when at least 75% of panelists assigned ratings of 4 or higher. If the initial voting failed to reach this threshold, the panel engaged to refine the recommendation language, followed by subsequent voting rounds employing a modified Delphi method. This systematic and multi-stage process ensured the production of evidence-based recommendations that incorporate diverse clinical expertise within Taiwan's healthcare context. The final recommendation statements were secured and released on 15/Sep/2025, fitting to the contemporary global evidence at the time point.

To achieve the expert consensus on the *Taiwan Consensus and Management Guidelines for Gastric Cancer*, the level of agreement (LoA) from each voting procedure was categorized as follows: (*****) 100% agreement, (****) 80–99% agreement, (***) 60–79% agreement, (**) 30–59% agreement, and (*) absence of agreement. Following consensus voting, the recommendation statements were developed based on a comprehensive review to determine the appropriate level of evidence (LoE). After justifying LoA and LoE, recommendation statements were adapted from the Grading of Recommendations Assessment, Development, and Evaluation (GRADE) methodology.

**Supplemental 4. Level of evidence and grade of recommendations among the included guidelines**

| **Guidelines** | **Level of evidence (LoE)** | **Grade of recommendations (GoR)** | **Note** |
| --- | --- | --- | --- |
| TWGCA 2025 (present guideline) | GRADE-adopted MINDS method | GRADE | Provides LoE, GoR and LoA in parallel |
| JGCA 2021 (7^th^) | GRADE-adopted MINDS method | For clinical questions:  consensus using MINDS method  For therapies:  GRADE method | LoE or GoR not consistently declared for non-therapy sections |
| KGCA 2024 | GRADE method | GRADE method | Meta-analyses for de novo issues |
| CSCO 2023 | Expert consensus-based system | Expert consensus-based system |  |
| NCCN 2026 (version 1) | NCCN Categories of Evidence and Consensus | No formal recommendation grades | 1. “Preferred” option(s) instead of recommendations  2. All suggestions are Category 2A unless otherwise specified |
| ESMO 2022, living guidelines 2024 (version 1.4) and Pan-Asian-adapted | IDSA-USPHS Grading System | IDSA-USPHS Grading System | ESMO-MCBS and ESCAT systems in conjunction |

LoE, level of evidence; GoR, grade of recommendations; LoA; level of agreement; TWGCA, Taiwan Gastric Cancer Association; JGCA, Japan Gastric Cancer Association; MINDS, Medical Information Network Distribution Service; GRADE, Grading of Recommendations Assessment, Development, and Evaluation; KGCA, Korean Gastric Cancer Association; CSCO, Chinese Society of Clinical Oncology; NCCN, National Comprehensive Cancer Network; ESMO, European Society for Medical Oncology; IDSA-USPHS, Infectious Diseases Society of America-United States Public Health Grading system; MCBS, Magnitude of Clinical Benefit Scale; ESCAT, ESMO Scale for Clinical Actionability of molecular Targets.

References:

1. Liou J-M, Malfertheiner P, Hong T-C, Cheng H-C, Sugano K, Shah S, et al. Screening and eradication of <em>Helicobacter pylori f</em>or gastric cancer prevention: Taipei Global Consensus II. Gut. 2025;74(11):1767-91. doi: 10.1136/gutjnl-2025-336027.

2. Kwee RM, Kwee TC. Imaging in local staging of gastric cancer: a systematic review. J Clin Oncol. 2007;25(15):2107-16. doi: 10.1200/JCO.2006.09.5224. PubMed PMID: 17513817.

3. Seevaratnam R, Cardoso R, McGregor C, Lourenco L, Mahar A, Sutradhar R, et al. How useful is preoperative imaging for tumor, node, metastasis (TNM) staging of gastric cancer? A meta-analysis. Gastric Cancer. 2012;15 Suppl 1:S3-18. Epub 20110812. doi: 10.1007/s10120-011-0069-6. PubMed PMID: 21837458.

4. Lee IJ, Lee JM, Kim SH, Shin CI, Lee JY, Kim SH, et al. Diagnostic performance of 64-channel multidetector CT in the evaluation of gastric cancer: differentiation of mucosal cancer (T1a) from submucosal involvement (T1b and T2). Radiology. 2010;255(3):805-14. doi: 10.1148/radiol.10091313. PubMed PMID: 20501718.

5. Mocellin S, Pasquali S. Diagnostic accuracy of endoscopic ultrasonography (EUS) for the preoperative locoregional staging of primary gastric cancer. The Cochrane database of systematic reviews. 2015;2015(2):Cd009944. Epub 2015/04/29. doi: 10.1002/14651858.CD009944.pub2. PubMed PMID: 25914908; PubMed Central PMCID: PMCPMC6465120.

6. Ajani JA, D'Amico TA, Bentrem DJ, Chao J, Cooke D, Corvera C, et al. Gastric Cancer, Version 2.2022, NCCN Clinical Practice Guidelines in Oncology. J Natl Compr Canc Netw. 2022;20(2):167-92. doi: 10.6004/jnccn.2022.0008. PubMed PMID: 35130500.

7. Kikuchi D, Iizuka T, Hoteya S, Yamada A, Furuhata T, Yamashita S, et al. Usefulness of magnifying endoscopy with narrow-band imaging for determining tumor invasion depth in early gastric cancer. Gastroenterol Res Pract. 2013;2013:217695. Epub 20130117. doi: 10.1155/2013/217695. PubMed PMID: 23401676; PubMed Central PMCID: PMCPMC3562685.

8. Yao K, Uedo N, Kamada T, Hirasawa T, Nagahama T, Yoshinaga S, et al. Guidelines for endoscopic diagnosis of early gastric cancer. Dig Endosc. 2020;32(5):663-98. doi: 10.1111/den.13684. PubMed PMID: 32275342.

9. Cardoso R, Coburn N, Seevaratnam R, Sutradhar R, Lourenco LG, Mahar A, et al. A systematic review and meta-analysis of the utility of EUS for preoperative staging for gastric cancer. Gastric Cancer. 2012;15 Suppl 1:S19-26. Epub 2012/01/13. doi: 10.1007/s10120-011-0115-4. PubMed PMID: 22237654.

10. Kim IH, Kang SJ, Choi W, Seo AN, Eom BW, Kang B, et al. Korean Practice Guidelines for Gastric Cancer 2024: An Evidence-based, Multidisciplinary Approach (Update of 2022 Guideline). Journal of gastric cancer. 2025;25(1):5-114. doi: 10.5230/jgc.2025.25.e11. PubMed PMID: 39822170; PubMed Central PMCID: PMCPMC11739648.

11. Spolverato G, Ejaz A, Kim Y, Squires MH, Poultsides GA, Fields RC, et al. Use of endoscopic ultrasound in the preoperative staging of gastric cancer: a multi-institutional study of the US gastric cancer collaborative. J Am Coll Surg. 2015;220(1):48-56. Epub 20140718. doi: 10.1016/j.jamcollsurg.2014.06.023. PubMed PMID: 25283742.

12. Tsendsuren T, Jun SM, Mian XH. Usefulness of endoscopic ultrasonography in preoperative TNM staging of gastric cancer. World journal of gastroenterology. 2006;12(1):43-7. doi: 10.3748/wjg.v12.i1.43. PubMed PMID: 16440415; PubMed Central PMCID: PMCPMC4077489.

13. Gotoda T, Yanagisawa A, Sasako M, Ono H, Nakanishi Y, Shimoda T, et al. Incidence of lymph node metastasis from early gastric cancer: estimation with a large number of cases at two large centers. Gastric Cancer. 2000;3(4):219-25. doi: 10.1007/pl00011720. PubMed PMID: 11984739.

14. Ono H, Yao K, Fujishiro M, Oda I, Uedo N, Nimura S, et al. Guidelines for endoscopic submucosal dissection and endoscopic mucosal resection for early gastric cancer (second edition). Dig Endosc. 2021;33(1):4-20. Epub 20201209. doi: 10.1111/den.13883. PubMed PMID: 33107115.

15. Abdelfatah MM, Barakat M, Lee H, Kim JJ, Uedo N, Grimm I, et al. The incidence of lymph node metastasis in early gastric cancer according to the expanded criteria in comparison with the absolute criteria of the Japanese Gastric Cancer Association: a systematic review of the literature and meta-analysis. Gastrointestinal endoscopy. 2018;87(2):338-47. Epub 20170928. doi: 10.1016/j.gie.2017.09.025. PubMed PMID: 28966062.

16. Kim HJ, Kim AY, Oh ST, Kim JS, Kim KW, Kim PN, et al. Gastric cancer staging at multi-detector row CT gastrography: comparison of transverse and volumetric CT scanning. Radiology. 2005;236(3):879-85. Epub 2005/07/16. doi: 10.1148/radiol.2363041101. PubMed PMID: 16020558.

17. Al-Batran SE, Homann N, Pauligk C, Goetze TO, Meiler J, Kasper S, et al. Perioperative chemotherapy with fluorouracil plus leucovorin, oxaliplatin, and docetaxel versus fluorouracil or capecitabine plus cisplatin and epirubicin for locally advanced, resectable gastric or gastro-oesophageal junction adenocarcinoma (FLOT4): a randomised, phase 2/3 trial. Lancet (London, England). 2019;393(10184):1948-57. Epub 2019/04/16. doi: 10.1016/s0140-6736(18)32557-1. PubMed PMID: 30982686.

18. Kang YK, Yook JH, Park YK, Lee JS, Kim YW, Kim JY, et al. PRODIGY: A Phase III Study of Neoadjuvant Docetaxel, Oxaliplatin, and S-1 Plus Surgery and Adjuvant S-1 Versus Surgery and Adjuvant S-1 for Resectable Advanced Gastric Cancer. J Clin Oncol. 2021;39(26):2903-13. Epub 2021/06/17. doi: 10.1200/jco.20.02914. PubMed PMID: 34133211; PubMed Central PMCID: PMCPMC8425847 Squibb, Zymeworks, ALX Oncology, Amgen, Novartis, MacroGenics, Surface Oncology Min-Hee RyuHonoraria: DAEHWA Pharmaceutical, Bristol-Myers Squibb, Lilly, Ono Pharmaceutical, MSD, Taiho Pharmaceutical, Novartis, Daiichi Sankyo, AstraZenecaConsulting or Advisory Role: DAEHWA Pharmaceutical, Bristol-Myers Squibb, Lilly, Ono Pharmaceutical, MSD, Taiho Pharmaceutical, Novartis, Daiichi Sankyo, AstraZeneca Sun Young RhaConsulting or Advisory Role: MSD Oncology, Ipsen, Daiichi Sankyo, Eisai, Amgen, IndivumedSpeakers' Bureau: Lilly, EisaiResearch Funding: MSD Oncology, Bristol-Myers Squibb, Eisai, Roche/Genentech, MedPacto, ASLAN Pharmaceuticals, SillaJen, Bayer, Immunomet Gyunji KimEmployment: Sanofi, NovartisStock and Other Ownership Interests: Sanofi YeonJu LeeEmployment: SanofiStock and Other Ownership Interests: Sanofi Jee Hyun LeeEmployment: SanofiNo other potential conflicts of interest were reported.

19. Zhang X, Liang H, Li Z, Xue Y, Wang Y, Zhou Z, et al. Perioperative or postoperative adjuvant oxaliplatin with S-1 versus adjuvant oxaliplatin with capecitabine in patients with locally advanced gastric or gastro-oesophageal junction adenocarcinoma undergoing D2 gastrectomy (RESOLVE): an open-label, superiority and non-inferiority, phase 3 randomised controlled trial. The Lancet Oncology. 2021;22(8):1081-92. Epub 2021/07/13. doi: 10.1016/s1470-2045(21)00297-7. PubMed PMID: 34252374.

20. Shitara K, Rha SY, Wyrwicz LS, Oshima T, Karaseva N, Osipov M, et al. Neoadjuvant and adjuvant pembrolizumab plus chemotherapy in locally advanced gastric or gastro-oesophageal cancer (KEYNOTE-585): an interim analysis of the multicentre, double-blind, randomised phase 3 study. The Lancet Oncology. 2024;25(2):212-24. Epub 2023/12/23. doi: 10.1016/s1470-2045(23)00541-7. PubMed PMID: 38134948.

21. Janjigian YY, Al-Batran S-E, Wainberg ZA, Muro K, Molena D, Cutsem EV, et al. Perioperative Durvalumab in Gastric and Gastroesophageal Junction Cancer. New England Journal of Medicine. 2025;393(3):217-30. doi: doi:10.1056/NEJMoa2503701.

22. Bonnot P-E, Piessen G, Kepenekian V, Decullier E, Pocard M, Meunier B, et al. Cytoreductive Surgery With or Without Hyperthermic Intraperitoneal Chemotherapy for Gastric Cancer With Peritoneal Metastases (CYTO-CHIP study): A Propensity Score Analysis. Journal of Clinical Oncology. 2019;37(23):2028-40. doi: 10.1200/jco.18.01688. PubMed PMID: 31084544.

23. Ishigami H, Fujiwara Y, Fukushima R, Nashimoto A, Yabusaki H, Imano M, et al. Phase III Trial Comparing Intraperitoneal and Intravenous Paclitaxel Plus S-1 Versus Cisplatin Plus S-1 in Patients With Gastric Cancer With Peritoneal Metastasis: PHOENIX-GC Trial. J Clin Oncol. 2018;36(19):1922-9. Epub 2018/05/11. doi: 10.1200/jco.2018.77.8613. PubMed PMID: 29746229.

24. Yarema RR, Ohorchak MA, Zubarev GP, Mylyan YP, Oliynyk YY, Zubarev MG, et al. Hyperthermic intraperitoneal chemoperfusion in combined treatment of locally advanced and disseminated gastric cancer: results of a single-centre retrospective study. International journal of hyperthermia : the official journal of European Society for Hyperthermic Oncology, North American Hyperthermia Group. 2014;30(3):159-65. Epub 2014/03/20. doi: 10.3109/02656736.2014.893451. PubMed PMID: 24641798.

25. National Comprehensive Cancer Network. Gastric Cancer (version 1, 2026). 2026;<https://www.nccn.org/professionals/physician_gls/pdf/gastric.pdf>.

26. Shitara K, Fleitas T, Kawakami H, Curigliano G, Narita Y, Wang F, et al. Pan-Asian adapted ESMO Clinical Practice Guidelines for the diagnosis, treatment and follow-up of patients with gastric cancer. ESMO open. 2024;9(2):102226. Epub 2024/03/09. doi: 10.1016/j.esmoop.2023.102226. PubMed PMID: 38458658; PubMed Central PMCID: PMCPMC10937212.

27. Kim I-H, Kang SJ, Choi W, Seo AN, Eom BW, Kang B, et al. Korean Practice Guidelines for Gastric Cancer 2024: An Evidence-based, Multidisciplinary Approach (Update of 2022 Guideline). Journal of gastric cancer. 2025;25(1):5-114.

28. Khalili-Tanha G, Khalili-Tanha N, Rouzbahani AK, Mahdieh R, Jasemi K, Ghaderi R, et al. Diagnostic, prognostic, and predictive biomarkers in gastric cancer: from conventional to novel biomarkers. Translational Research. 2024;274:35-48. doi: <https://doi.org/10.1016/j.trsl.2024.09.001>.

29. Okazaki U, Nakayama I, Sakamoto N, Kuwata T, Kawazoe A, Yoshida M, et al. Clinical implementation of simultaneous multiple biomarkers testing for&#xa0;metastatic or recurrent gastroesophageal adenocarcinoma: a single-institutional experience. ESMO Gastrointestinal Oncology. 2024;5. doi: 10.1016/j.esmogo.2024.100086.

30. Nakamura Y, Shitara K, Lee J. The Right Treatment of the Right Patient: Integrating Genetic Profiling Into Clinical Decision Making in Advanced Gastric Cancer in Asia. American Society of Clinical Oncology Educational Book. 2021(41):e166-e73. doi: 10.1200/edbk_321247. PubMed PMID: 34010049.

31. Bang YJ, Van Cutsem E, Feyereislova A, Chung HC, Shen L, Sawaki A, et al. Trastuzumab in combination with chemotherapy versus chemotherapy alone for treatment of HER2-positive advanced gastric or gastro-oesophageal junction cancer (ToGA): a phase 3, open-label, randomised controlled trial. Lancet (London, England). 2010;376(9742):687-97. Epub 2010/08/24. doi: 10.1016/s0140-6736(10)61121-x. PubMed PMID: 20728210.

32. Janjigian YY, Kawazoe A, Bai Y, Xu J, Lonardi S, Metges JP, et al. Pembrolizumab plus trastuzumab and chemotherapy for HER2-positive gastric or gastro-oesophageal junction adenocarcinoma: interim analyses from the phase 3 KEYNOTE-811 randomised placebo-controlled trial. Lancet (London, England). 2023;402(10418):2197-208. Epub 2023/10/24. doi: 10.1016/s0140-6736(23)02033-0. PubMed PMID: 37871604.

33. Janjigian YY, Kawazoe A, Yañez P, Li N, Lonardi S, Kolesnik O, et al. The KEYNOTE-811 trial of dual PD-1 and HER2 blockade in HER2-positive gastric cancer. Nature. 2021;600(7890):727-30. Epub 2021/12/17. doi: 10.1038/s41586-021-04161-3. PubMed PMID: 34912120; PubMed Central PMCID: PMCPMC8959470.

34. Rha SY, Oh DY, Yañez P, Bai Y, Ryu MH, Lee J, et al. Pembrolizumab plus chemotherapy versus placebo plus chemotherapy for HER2-negative advanced gastric cancer (KEYNOTE-859): a multicentre, randomised, double-blind, phase 3 trial. The Lancet Oncology. 2023;24(11):1181-95. Epub 2023/10/25. doi: 10.1016/s1470-2045(23)00515-6. PubMed PMID: 37875143.

35. Janjigian YY, Shitara K, Moehler M, Garrido M, Salman P, Shen L, et al. First-line nivolumab plus chemotherapy versus chemotherapy alone for advanced gastric, gastro-oesophageal junction, and oesophageal adenocarcinoma (CheckMate 649): a randomised, open-label, phase 3 trial. Lancet (London, England). 2021;398(10294):27-40. Epub 2021/06/09. doi: 10.1016/s0140-6736(21)00797-2. PubMed PMID: 34102137.

36. Kang YK, Chen LT, Ryu MH, Oh DY, Oh SC, Chung HC, et al. Nivolumab plus chemotherapy versus placebo plus chemotherapy in patients with HER2-negative, untreated, unresectable advanced or recurrent gastric or gastro-oesophageal junction cancer (ATTRACTION-4): a randomised, multicentre, double-blind, placebo-controlled, phase 3 trial. The Lancet Oncology. 2022;23(2):234-47. Epub 2022/01/15. doi: 10.1016/s1470-2045(21)00692-6. PubMed PMID: 35030335.

37. Qiu MZ, Oh DY, Kato K, Arkenau T, Tabernero J, Correa MC, et al. Tislelizumab plus chemotherapy versus placebo plus chemotherapy as first line treatment for advanced gastric or gastro-oesophageal junction adenocarcinoma: RATIONALE-305 randomised, double blind, phase 3 trial. BMJ (Clinical research ed). 2024;385:e078876. Epub 2024/05/29. doi: 10.1136/bmj-2023-078876. PubMed PMID: 38806195.

38. Shitara K, Lordick F, Bang Y-J, Enzinger P, Ilson D, Shah MA, et al. Zolbetuximab plus mFOLFOX6 in patients with CLDN18.2-positive, HER2-negative, untreated, locally advanced unresectable or metastatic gastric or gastro-oesophageal junction adenocarcinoma (SPOTLIGHT): a multicentre, randomised, double-blind, phase 3 trial. The Lancet. 2023;401(10389):1655-68. doi: 10.1016/S0140-6736(23)00620-7.

39. Shah MA, Shitara K, Ajani JA, Bang Y-J, Enzinger P, Ilson D, et al. Zolbetuximab plus CAPOX in CLDN18.2-positive gastric or gastroesophageal junction adenocarcinoma: the randomized, phase 3 GLOW trial. Nature medicine. 2023;29(8):2133-41. doi: 10.1038/s41591-023-02465-7.

40. Doebele RC, Drilon A, Paz-Ares L, Siena S, Shaw AT, Farago AF, et al. Entrectinib in patients with advanced or metastatic NTRK fusion-positive solid tumours: integrated analysis of three phase 1-2 trials. The Lancet Oncology. 2020;21(2):271-82. Epub 2019/12/16. doi: 10.1016/s1470-2045(19)30691-6. PubMed PMID: 31838007; PubMed Central PMCID: PMCPMC7461630.

41. Salama AKS, Li S, Macrae ER, Park JI, Mitchell EP, Zwiebel JA, et al. Dabrafenib and Trametinib in Patients With Tumors With BRAF(V600E) Mutations: Results of the NCI-MATCH Trial Subprotocol H. J Clin Oncol. 2020;38(33):3895-904. Epub 2020/08/08. doi: 10.1200/jco.20.00762. PubMed PMID: 32758030; PubMed Central PMCID: PMCPMC7676884 not necessarily represent the official views of the National Institutes of Health, nor does mention of trade names, commercial products, or organizations imply endorsement by the US government.

42. Subbiah V, Wolf J, Konda B, Kang H, Spira A, Weiss J, et al. Tumour-agnostic efficacy and safety of selpercatinib in patients with RET fusion-positive solid tumours other than lung or thyroid tumours (LIBRETTO-001): a phase 1/2, open-label, basket trial. The Lancet Oncology. 2022;23(10):1261-73. Epub 2022/09/16. doi: 10.1016/s1470-2045(22)00541-1. PubMed PMID: 36108661; PubMed Central PMCID: PMCPMC11702314.

43. Chung HC, Kang YK, Chen Z, Bai Y, Wan Ishak WZ, Shim BY, et al. Pembrolizumab versus paclitaxel for previously treated advanced gastric or gastroesophageal junction cancer (KEYNOTE-063): A randomized, open-label, phase 3 trial in Asian patients. Cancer. 2022;128(5):995-1003. Epub 2021/12/09. doi: 10.1002/cncr.34019. PubMed PMID: 34878659; PubMed Central PMCID: PMCPMC9299889.

44. Marabelle A, O’Malley DM, Hendifar AE, Ascierto PA, Motola-Kuba D, Penel N, et al. Pembrolizumab in microsatellite-instability-high and mismatch-repair-deficient advanced solid tumors: updated results of the KEYNOTE-158 trial. Nature Cancer. 2025;6(2):253-8. doi: 10.1038/s43018-024-00894-y.

45. Van Cutsem E, di Bartolomeo M, Smyth E, Chau I, Park H, Siena S, et al. Trastuzumab deruxtecan in patients in the USA and Europe with HER2-positive advanced gastric or gastroesophageal junction cancer with disease progression on or after a trastuzumab-containing regimen (DESTINY-Gastric02): primary and updated analyses from a single-arm, phase 2 study. The Lancet Oncology. 2023;24(7):744-56. Epub 2023/06/18. doi: 10.1016/s1470-2045(23)00215-2. PubMed PMID: 37329891; PubMed Central PMCID: PMCPMC11298287.

46. Shitara K, Cutsem EV, Gümüş M, Lonardi S, Fouchardière Cdl, Coutzac C, et al. Trastuzumab Deruxtecan or Ramucirumab plus Paclitaxel in Gastric Cancer. New England Journal of Medicine. 2025;393(4):336-48. doi: doi:10.1056/NEJMoa2503119.

47. Bonnot PE, Piessen G, Kepenekian V, Decullier E, Pocard M, Meunier B, et al. Cytoreductive Surgery With or Without Hyperthermic Intraperitoneal Chemotherapy for Gastric Cancer With Peritoneal Metastases (CYTO-CHIP study): A Propensity Score Analysis. J Clin Oncol. 2019;37(23):2028-40. Epub 2019/05/16. doi: 10.1200/jco.18.01688. PubMed PMID: 31084544.

48. Yang XJ, Huang CQ, Suo T, Mei LJ, Yang GL, Cheng FL, et al. Cytoreductive surgery and hyperthermic intraperitoneal chemotherapy improves survival of patients with peritoneal carcinomatosis from gastric cancer: final results of a phase III randomized clinical trial. Annals of surgical oncology. 2011;18(6):1575-81. Epub 2011/03/25. doi: 10.1245/s10434-011-1631-5. PubMed PMID: 21431408; PubMed Central PMCID: PMCPMC3087875.

49. Rau B, Lang H, Koenigsrainer A, Gockel I, Rau HG, Seeliger H, et al. Effect of Hyperthermic Intraperitoneal Chemotherapy on Cytoreductive Surgery in Gastric Cancer With Synchronous Peritoneal Metastases: The Phase III GASTRIPEC-I Trial. J Clin Oncol. 2024;42(2):146-56. Epub 2023/10/31. doi: 10.1200/jco.22.02867. PubMed PMID: 37906724; PubMed Central PMCID: PMCPMC10824373 manuscript. All relationships are considered compensated unless otherwise noted. Relationships are self-held unless noted. I = Immediate Family Member, Inst = My Institution. Relationships may not relate to the subject matter of this manuscript. For more information about ASCO's conflict of interest policy, please refer to [www.asco.org/rwc](file:///C:\Users\yssha\Downloads\www.asco.org\rwc) or ascopubs.org/jco/authors/author-center. Open Payments is a public database containing information reported by companies about payments made to US-licensed physicians (Open Payments).

50. Fujitani K, Yang HK, Mizusawa J, Kim YW, Terashima M, Han SU, et al. Gastrectomy plus chemotherapy versus chemotherapy alone for advanced gastric cancer with a single non-curable factor (REGATTA): a phase 3, randomised controlled trial. The Lancet Oncology. 2016;17(3):309-18. Epub 2016/01/30. doi: 10.1016/s1470-2045(15)00553-7. PubMed PMID: 26822397.

51. Yoshida K, Yasufuku I, Terashima M, Young Rha S, Moon Bae J, Li G, et al. International Retrospective Cohort Study of Conversion Therapy for Stage IV Gastric Cancer 1 (CONVO-GC-1). Annals of gastroenterological surgery. 2022;6(2):227-40. Epub 2022/03/10. doi: 10.1002/ags3.12515. PubMed PMID: 35261948; PubMed Central PMCID: PMCPMC8889854.

52. Al-Batran S-E, Homann N, Pauligk C, Illerhaus G, Martens UM, Stoehlmacher J, et al. Effect of Neoadjuvant Chemotherapy Followed by Surgical Resection on Survival in Patients With Limited Metastatic Gastric or Gastroesophageal Junction Cancer: The AIO-FLOT3 Trial. JAMA Oncology. 2017;3(9):1237-44. doi: 10.1001/jamaoncol.2017.0515.

53. Al-Batran SE, Goetze TO, Mueller DW, Vogel A, Winkler M, Lorenzen S, et al. The RENAISSANCE (AIO-FLOT5) trial: effect of chemotherapy alone vs. chemotherapy followed by surgical resection on survival and quality of life in patients with limited-metastatic adenocarcinoma of the stomach or esophagogastric junction - a phase III trial of the German AIO/CAO-V/CAOGI. BMC cancer. 2017;17(1):893. Epub 2017/12/29. doi: 10.1186/s12885-017-3918-9. PubMed PMID: 29282088; PubMed Central PMCID: PMCPMC5745860.

54. Kim TH, Uyama I, Rha SY, Bencivenga M, An J, Wyrwicz L, et al. Conversion Therapy for Stage IV Gastric Cancer: Report From the Expert Consensus Meeting at KINGCA WEEK 2024. Journal of gastric cancer. 2025;25(1):133-52. Epub 2025/01/17. doi: 10.5230/jgc.2025.25.e9. PubMed PMID: 39822172; PubMed Central PMCID: PMCPMC11739646.
